# Human adipose tissue as a major reservoir of cytomegalovirus-reactive T cells

**DOI:** 10.3389/fimmu.2023.1303724

**Published:** 2023-11-20

**Authors:** Anaïs Redruello-Romero, Maria S. Benitez-Cantos, David Lopez-Perez, Jesús García-Rubio, Francisco Tamayo, Daniel Pérez-Bartivas, Sara Moreno-SanJuan, Isabel Ruiz-Palmero, Jose D. Puentes-Pardo, Jose R. Vilchez, Miguel Á. López-Nevot, Federico García, Carlos Cano, Josefa León, Ángel Carazo

**Affiliations:** ^1^ Research Unit, Biosanitary Research Institute of Granada (ibs.GRANADA), Granada, Spain; ^2^ Department of Biochemistry and Molecular Biology III and Immunology, Faculty of Medicine, University of Granada, Granada, Spain; ^3^ GENYO, Centre for Genomics and Oncological Research: Pfizer/University of Granada/Andalusian Regional Government, Granada, Spain; ^4^ Department of Pharmacology, Faculty of Pharmacy, University of Granada, Granada, Spain; ^5^ Surgery Unit, San Cecilio University Hospital, Granada, Spain; ^6^ Cytometry and Microscopy Research Service, Biosanitary Research Institute of Granada (ibs.GRANADA), Granada, Spain; ^7^ Clinical Analyses and Immunology Unit, Virgen de las Nieves University Hospital, Granada, Spain; ^8^ Clinical Microbiology Unit, San Cecilio University Hospital, Granada, Spain; ^9^ Centro de Investigación Biomédica en Red (CIBER) of Infectious Diseases, Health Institute Carlos III, Madrid, Spain; ^10^ Department of Computer Science and Artificial Intelligence, University of Granada, Granada, Spain; ^11^ Digestive Unit, San Cecilio University Hospital, Granada, Spain

**Keywords:** T cell receptor, adipose tissue, cytomegalovirus, tissue-resident memory T cells, antigen prediction

## Abstract

**Introduction:**

Cytomegalovirus (CMV) is a common herpesvirus with a high prevalence worldwide. After the acute infection phase, CMV can remain latent in several tissues. CD8 T cells in the lungs and salivary glands mainly control its reactivation control. White adipose tissue (WAT) contains a significant population of memory T cells reactive to viral antigens, but CMV specificity has mainly been studied in mouse WAT. Therefore, we obtained blood, omental WAT (oWAT), subcutaneous WAT (sWAT), and liver samples from 11 obese donors to characterize the human WAT adaptive immune landscape from a phenotypic and immune receptor specificity perspective.

**Methods:**

We performed high-throughput sequencing of the T cell receptor (TCR) locus to analyze tissue and blood TCR repertoires of the 11 donors. The presence of TCRs specific to CMV epitopes was tested through ELISpot assays. Moreover, phenotypic characterization of T cells was carried out through flow cytometry.

**Results:**

High-throughput sequencing analyses revealed that tissue TCR repertoires in oWAT, sWAT, and liver samples were less diverse and dominated by hyperexpanded clones when compared to blood samples. Additionally, we predicted the presence of TCRs specific to viral epitopes, particularly from CMV, which was confirmed by ELISpot assays. Remarkably, we found that oWAT has a higher proportion of CMV-reactive T cells than blood or sWAT. Finally, flow cytometry analyses indicated that most WAT-infiltrated lymphocytes were tissue-resident effector memory CD8 T cells.

**Discussion:**

Overall, these findings postulate human oWAT as a major reservoir of CMV-specific T cells, presumably for latent viral reactivation control. This study enhances our understanding of the adaptive immune response in human WAT and highlights its potential role in antiviral defense.

## 1 Introduction

Cytomegalovirus (CMV) is a common type of herpesvirus that has a high prevalence in the population, infecting 50-90% of adults in developing countries (1). In most cases, patients are asymptomatic. In immunocompetent conditions, the viral reactivation is controlled by blood-circulating and tissue-resident leukocytes, particularly by CD8+ T cells (2). However, infections in immunocompromised individuals can lead to severe conditions and even death (3). In this context, studies of latent infection control among the body have pinpointed adipose tissue as a relevant reservoir of adaptive immune cells for lifelong control of CMV infection (4, 5).

The understanding of white adipose tissue (WAT) has noticeably evolved. Initially regarded as a mere storage of neutral lipids, it is currently considered an endocrine organ, essential in regulating systemic energy homeostasis (6). In addition, WAT plays a role in non-shivering thermogenesis by the generation of a particular kind of thermogenic adipocyte (7). This multi-functional vision of WAT is still changing since a new role as an immune organ is emerging (8).

Different cell types occur in WAT, including white and thermogenic adipocytes, fibroblasts, endothelial cells, different populations of stem cells and a wide variety of leukocytes (from both, adaptive and innate immune systems) (9). They all contribute to the main functions of WAT: energy storage, endocrine regulation, non-shivering thermogenesis, and host defense. Therefore, WAT is currently seen as a complex tissue full of synergistic interactions between different cell types (8, 10–13).

WAT contains a lymphoid structure called fat-associated lymphoid clusters (FALCs) or milky spots (8, 14, 15). Regarding immune defense, FALCs resemble secondary or tertiary lymphoid organs. They are particularly interesting in the peritoneal cavity since they filter peritoneal fluid and capture soluble antigens (16). When microbial antigens are detected, fibroblastic reticular cells in FALCs recruit peripheral monocytes and promote the formation of new FALCs. This is followed by rapid activation of T and B cells.

FALCs contain a huge amount of memory T cells (8, 9, 17). Most of them belong to the resident memory compartment and do not recirculate. This accumulation of tissue-resident memory T cells (Trm) in FALCs has two advantages. Firstly, WAT connects all organs with lymphoid structures and, during inflammation, lymph leak from vessels reaching WAT lymphatics and FALCs. Therefore, this mechanism works synergistically with classic lymphatic drainage. Secondly, the main source of energy in Trm is mitochondrial fatty acid oxidation (17). Interestingly, upon microbial challenge, adipocytes increase lipolysis making fatty acids available to Trm (13). In a nutshell, T cells in FALCs are located in a strategic location full of fuel to respond to microbes’ reencounter. Besides, CD8+ Trm in WAT shows a higher proliferative potential than Trm in other locations and co-expresses IFN-γ and TNF-alfa 1h after activation (17).

A fraction of WAT-resident memory T cells has been demonstrated to be reactive to viral antigens (17, 18). At present, only a few works (mainly in murine models) have studied the WAT reservoir of viral-specific memory T cells (4, 17). Murine CMV-specific CD8 T cells have been identified in mice adipose tissue and have been associated with the control of lifelong infection (4). Some studies in humans have described the role of a minor subpopulation of CMV-specific CD4+ T cells in sWAT of patients coinfected with human immunodeficiency viruses (HIV) and CMV (5), and the presence of Trm cells reactive to CMV antigens in respiratory mucosa (19).

The antigen specificity of a T cell is determined by its T cell receptor (TCR) sequence. High-throughput sequencing of the TCR repertoire can provide a snapshot of the clonal expansion level of an immune reservoir, as well as its antigen specificities if a given TCR has a known, experimentally determined, cognate antigen. In the present work we have generated TCR libraries of 11 donors from blood, omental WAT (oWAT), subcutaneous WAT (sWAT) and liver to study the adaptive immunity landscape of tissue reservoirs. We found T cells annotated to be specific to common viral pathogens in WAT, specially to CMV antigens. The presence of CMV-specific T cells in human WAT was confirmed by ELISpot assays in all CMV-seropositive donors. Moreover, we characterized the phenotype of WAT-infiltrating T cells by flow cytometry. The fact that tissue-resident effector memory T cells (Tem) are the most abundant population in WAT, together with the enrichment in CMV-specific T lymphocytes, supports the hypothesis that WAT contains an immunological reservoir responsible for controlling reactivation of latent CMV in humans.

## 2 Results

### 2.1 Baseline characteristics of patients

**Table 1** shows details on data of age, sex, type 2 diabetes (T2D), hypertension, body mass index (BMI), glucose, glycated hemoglobin (HbA1c), C-reactive protein (CRP), cholesterol, triglycerides, high-density lipoprotein (HDL), low-density lipoprotein (LDL), CMV and EBV (Epstein-Barr virus) seropositivity. Women were predominant, around 64% of patients, due to social constraints in our geographic environment.

**Table 1:** Clinical baseline characteristics of patients.

Variables	Patient										
	#1	#2	#3	#4	#5	#6	#7	#8	#9	#10	#11
Age (years)	37	36	55	55	53	50	44	65	38	48	55
Female/Male	F	F	M	M	F	M	F	F	M	F	F
T2D	+	–	–	+	–	+	–	–	–	–	–
Hypertension	+	–	+	–	+	+	–	+	–	–	+
BMI (kg/m2)	38.2	39	33.8	50	36.9	40.6	45.5	37.1	48.1	41.8	42.8
Glucose (mg/dL)	151	88	75	98	92	318	116	88	90	105	101
Hb1Ac (%)	5.5.	5	5.7	7	5.4	5.5	5.7	6.2	5.4	5.5	5.5
CRP (mg/L)	18.7	5	9.3	22	11.9	2.7	20.9	5.9	5.3	4.1	16.6
Cholesterol (mg/dL)	128	118	156	125	125	201	131	143	143	188	149
Triglycerides (mg/dL)	175	156	104	105	48	12	202	175	85	264	120
HDL (mg/dL)	39	29	39	41	61	34	27	52	35	34	38
LDL (mg/dL)	54	58	96	63	80	134	64	56	91	101	87
CMV (IgG/IgM)	+/-	+/-	+/-	+/-	+/-	+/-	+/-	+/-	-/-	+/-	+/-
EBV (IgG/IgM)	+/-	+/-	+/-	+/-	+/-	+/-	+/-	+/-	-/-	+/-	+/-

### 2.2 TCR repertoire analysis

In order to study the immune receptor landscape in different tissue compartments, we assessed TCRβ repertoire diversity and overlap in obese patients from blood, oWAT, sWAT, and liver. It must be noted that liver samples were exclusively used for TCRβ analyses due to the small size of hepatic biopsies. We obtained a total of 5 million reads covering full V-D-J regions (400-600 bp long). After identifying the V and J alleles and the complementary determining region 3 (CDR3) sequence, we found 86,005 unique TCRs among our samples.

**Figure 1:**
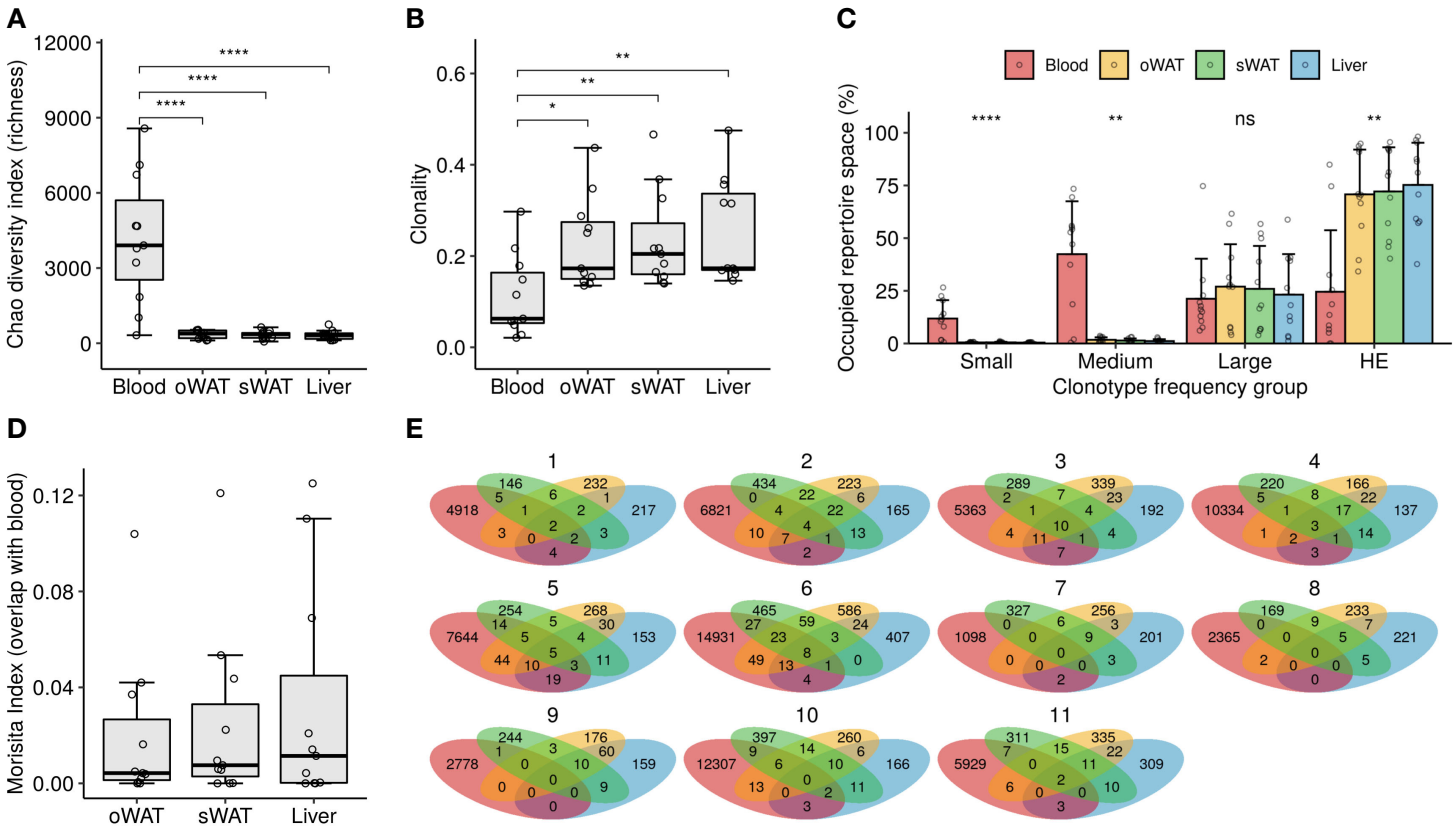
TCR repertoire diversity measurements. **(A)** Richness analysis (estimation of the number of different TCRs in a sample). **(B)** Clonality degree of the repertoires. **(C)** Percentage of occupied repertoire space by clones of a given size (i.e., relative frequency in the repertoire): small (≤ 0.0001), medium (0.0001 – 0.001), large (0.001 – 0.01) and hyperexpanded (HE, > 0.01). Statistical differences among tissues within each group were assessed with Kruskal-Wallis test. **(D)** Degree of overlap of the solid tissue repertoires with their respective blood repertoires. **(E)** Venn diagrams depicting the number of shared clonotypes among repertoires from the same patient. ns: non-significant, *: p ≤ 0.05, **: p ≤ 0.01, ****: p ≤ 0.0001.

Chao index was calculated to measure repertoire richness, understood as the number of different clonotypes in a sample. Blood repertoires showed a significantly higher richness compared to solid tissue repertoires (4201 ± 2556 clonotypes in blood, 343 ± 166 in oWAT, 441 ± 181 in sWAT and 296 ± 173 in liver, Wilcoxon test, p < 0.0001) (**Figure 1A**). Clonality was calculated to measure the degree of clonal expansion in a TCR repertoire. We found that solid tissue samples are significantly more clonal than blood repertoires (0.11 ± 0.09 in blood, 0.23 ± 0.10 in oWAT, 0.24 ± 0.11 in sWAT and 0.26 ± 0.11 in liver, Wilcoxon test, p < 0.05) (**Figure 1B**). No significant differences in richness and clonality were observed between solid tissues. Additionally, we studied clonality by assessing the clonal space homeostasis, i.e., the proportion of the repertoire that is occupied by clonotypes of a given frequency. Four different groups were established: small (clonotype frequency ≤ 0.0001), medium (0.0001-0.001), large (0.001-0.01) and hyperexpanded (>0.01). Blood repertoires were mainly composed of medium clones, whereas solid tissues were dominated by hyperexpanded lymphocytes (**Figure 1C**). Altogether, these findings indicate that tissue infiltrating TCR repertoires were less diverse and more expanded compared to the set of circulating blood lymphocytes.

Overlap between blood and tissue repertoires was assessed with the Morisita index, where 0 meant that the samples do not have any clonotypes in common and 1 meant that all the TCRs occur in the same proportion in the two repertoires compared. Low overlap with blood was observed for all the solid tissues since the maximum overlap value observed was 0.13 in the blood-liver comparison of patient #1 (**Figure 1D**). No significant differences were observed between solid tissues in the degree of overlap with blood repertoires (Kruskal-Wallis test, p > 0.05). The number of shared clonotypes between tissues of the same patient is shown in **Figure 1E**. Again, little to no overlap is observed among different TCR repertoires from the same patient, indicating that the subsets of tissue-infiltrated clonotypes are very distinct and that some of them are even undetectable in blood.

### 2.3 Antigen specificity prediction

To predict the antigen specificity of TCR repertoires we accessed the VDJdb database, which contains curated TCR sequences with *in vitro* determined antigen specificity that were used to annotate our repertoires by sequence similarity clustering. Of the 86,005 TCRs discovered in our repertoires, only 363 clustered with any CDR3 sequence of VDJdb, leaving 99.58% of our data unannotated for antigen specificity. This highlights the need to perform more *in vitro* antigen specificity assays to enlarge these databases.

**Figure 2:**
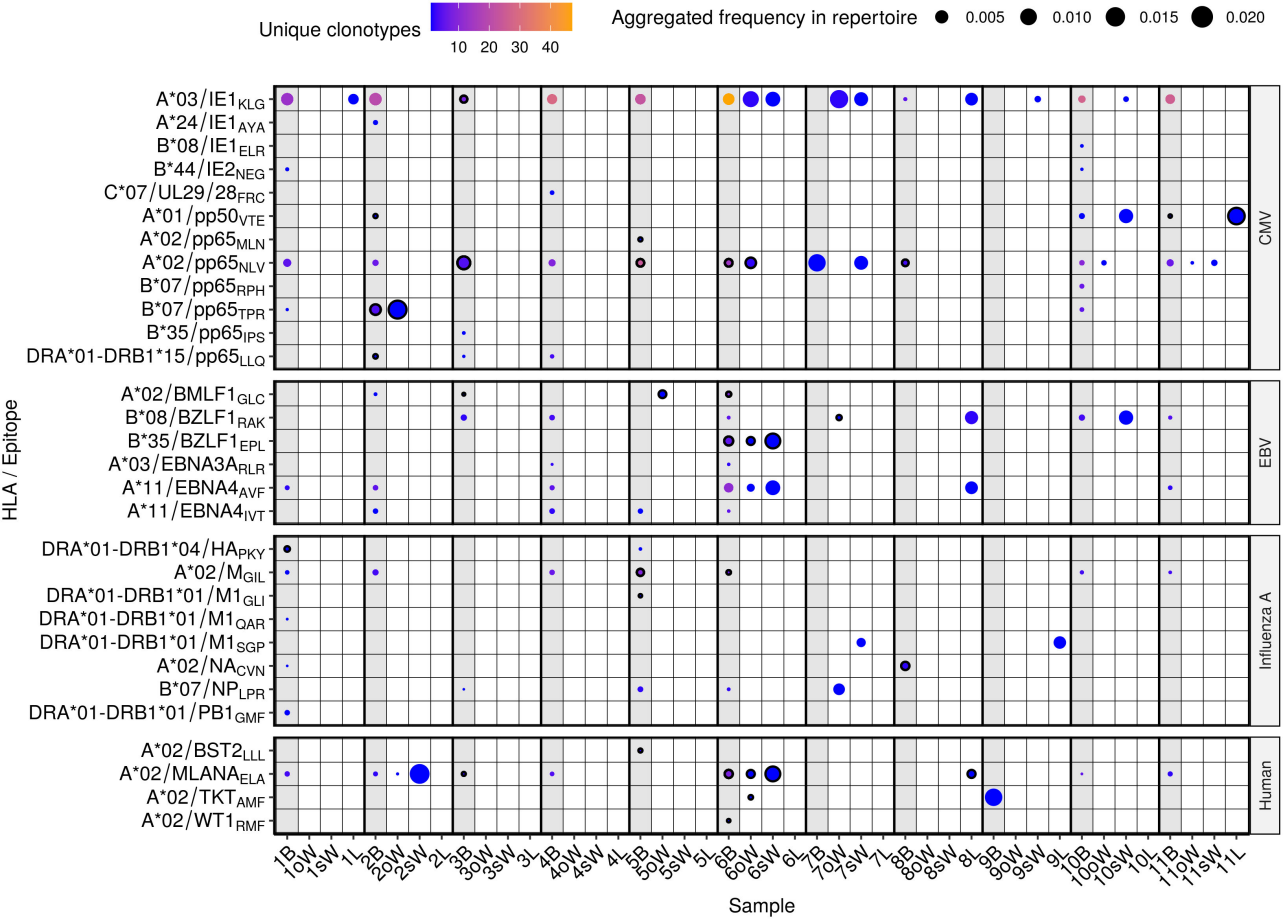
Predicted antigen specificity of the 44 TCR repertoires analyzed. Epitopes (y axis) are identified by the HLA allele presenting them, the protein of origin and their first three amino acids. Each circle represents the specificity of TCRs to each epitope in a given repertoire. Color scale stands for the number of different clonotypes recognizing that epitope, while size represents the relative frequency of those clonotypes in the repertoire. Blood samples from each donor are highlighted in light grey and outlined circles indicate that the patient carries the HLA allele presenting the epitope according to the VDJdb database. B, blood; oW, omental white adipose tissue; sW, subcutaneous white adipose tissue; L, liver tissue.

Specificity to 30 epitopes from 19 antigens was found in our repertoires, both in blood and in solid tissues, including several viral epitopes from CMV, EBV and Influenza virus A (**Figure 2**, **Supplementary Dataset 1**). It must be noted that 24 of the 30 epitopes are presented by HLA class I and 6 by HLA class II. Moreover, none of the latter epitopes were present in oWAT. For each TCR/epitope pair, the VDJdb database provides information about the HLA allele that was used in the antigen specificity assay. However, due to the known promiscuity of HLA alleles in antigen presentation (20, 21), we showed all the antigen specificities discovered, even when the HLA allele reported in the database is not carried by our patients.

TCRs specific to 8 CMV epitopes were present exclusively in blood samples, while specificity to 4 epitopes was found both in blood and WAT in 5 out of 11 patients (45.5%). A*03/IE1_KLG_ was predicted to be the most widely recognized CMV epitope in our cohort, with specific TCRs present in oWAT from patient #6 and sWAT from patients #6, #7, #9 and #10. It should be mentioned that it is the epitope with the largest number of known TCRs in the VDJdb database, which may bias the results. A*02/pp65_NLV_ had cognate TCRs in all the patients but patient #9, which was consistent with them being the only patient who tested negative in CMV serology. Specificity against this antigen was predicted in oWAT from patients #6 (carrier of the HLA-A*02 allele, **Supplementary Table 1**), #10 and #11, and sWAT from patients #7 and #11. TCRs against another epitope from the CMV protein pp65, B*07/pp65_TPR_, were found in oWAT from patient #2, which also carries the HLA-B*07 allele, occupying around 16.8% of the sequenced repertoire. Specificity to EBV epitopes was found to a lesser extent in blood and solid tissues. Particularly, TCRs specific to B*35/BZLF1_EPL_ and A*11/EBNA4_AVF_ epitopes were found in oWAT and sWAT from patient #6, which showed the broadest specificity against EBV epitopes of the entire cohort. Epitopes from Influenza A were predicted to be recognized mostly by TCRs from circulating T lymphocytes. Only patient #7 showed specificity against epitopes B*07/NP_LPR_ and DRA*01-DRB1*01/M1_SGP_ in oWAT and sWAT, respectively. Specificity to self-antigens was also predicted in our cohort. Especially to A*02/MLANA_ELA_, derived from the melanoma antigen recognized by T cells 1 (known as MART1 or MLANA) in patients #2 and #6, which showed specific TCRs in blood, oWAT, but especially in sWAT, occupying 21.1% and 10.7% of the sequenced sWAT repertoire, respectively. Reactive T cells against MLANA appear in some pathological contexts, such as vitiligo and melanoma (22). Interestingly, patient #6 has been diagnosed with basal cell carcinoma afterwards.

### 2.4 ELISpot assay

**Figure 3:**
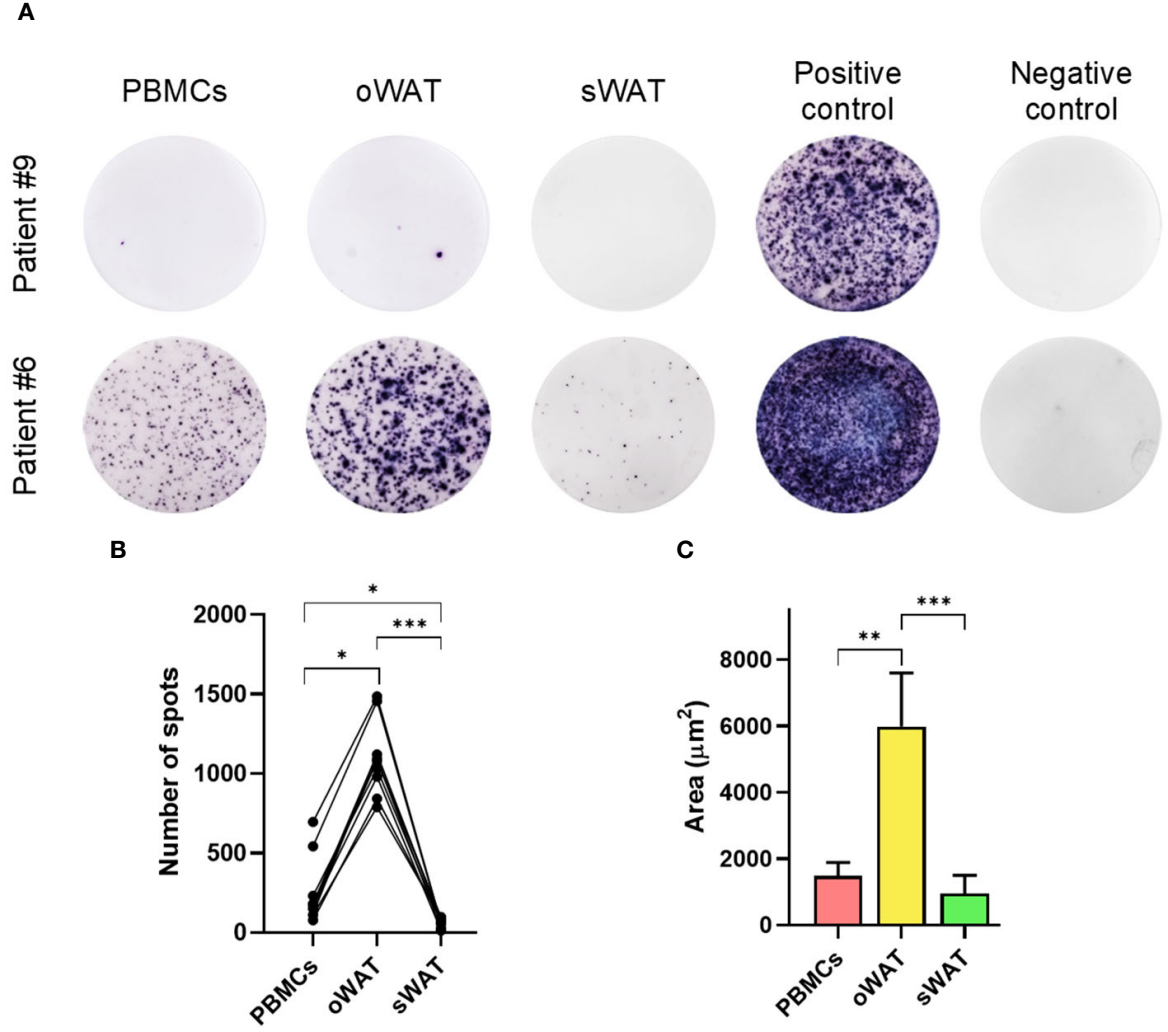
Cytomegalovirus ELISpot assays measurements. **(A)** Patient #9, negative for CMV and patient #6, positive for CMV in PBMCs and both tissues (oWAT and sWAT). **(B)** Total number of spots for all the different tissues in all CMV-positive patients. **(C)** First decile of the biggest spots for all the different tissues in all CMV-positive patients. *: p ≤ 0.05, **: p ≤ 0.01, ***: p ≤ 0.001.

One of the most notable results of the *in silico* antigen-specificity prediction was the relative abundance of CMV-specific TCRs in oWAT. To validate this result, we performed a functionality test with isolated T lymphocytes. ELISpot assays were carried out on every patient and results are shown in **Figure 3A** and **Supplementary Figure 1**.

The mean number of CMV reactive cells in oWAT was one order of magnitude more elevated than in peripheral blood mononuclear cells (PBMCs) (1174.45 ± 207.86 in oWAT *versus* 255.09 ± 194.37 in PBMCs, p<0.001, **Figure 3B**). In contrast, sWAT showed a reduced number of spots (45.36 ± 15.94). We must remark that the same number of mononuclear cells, either from blood or adipose tissue, was seeded in each well to perform the ELISpot assay. According to a previous article published by our group (9), the mononuclear set of stromal vascular fraction (SVF) is composed of 40-50% of T lymphocytes (in both, oWAT and sWAT). The mononuclear fraction of total blood is usually composed of 40-70% of T cells. In case of the study cohort, according to our cytometric data, these percentages are limited to 45-55% in all types of samples. Another remarkable difference was in the size of spots, which can be used as a measure of the amount of interferon-γ (IFN-γ) that is secreted by cells after TCR stimulation. In oWAT, a noticeable fraction of cells (around 16%) showed a high-secretion (spot area >2000µm^2^) level of IFN-γ, while this fraction is marginal in blood (around 2%) and absent in sWAT. In addition, as shown in **Figure 3C**, the first decile of oWAT had spot sizes significantly larger than sWAT and blood.

### 2.5 Phenotypic analyses of T cells by flow cytometry

In order to analyze the phenotype of T lymphocytes in blood, oWAT and sWAT, two different cytometric panels were used. Panel 1 included markers to identify the naïve phenotype (CCR7+/CD45RA+) and three memory populations: T central memory (Tcm: CCR7+/CD45RA-), T effector memory (Tem: CCR7-/CD45RA-) and T effector memory re-expressing CD45RA (Temra: CCR7-/CD45RA+). Panel 2 included several tissue-residence markers: CD69, CD49a and CD103.

**Figure 4:**
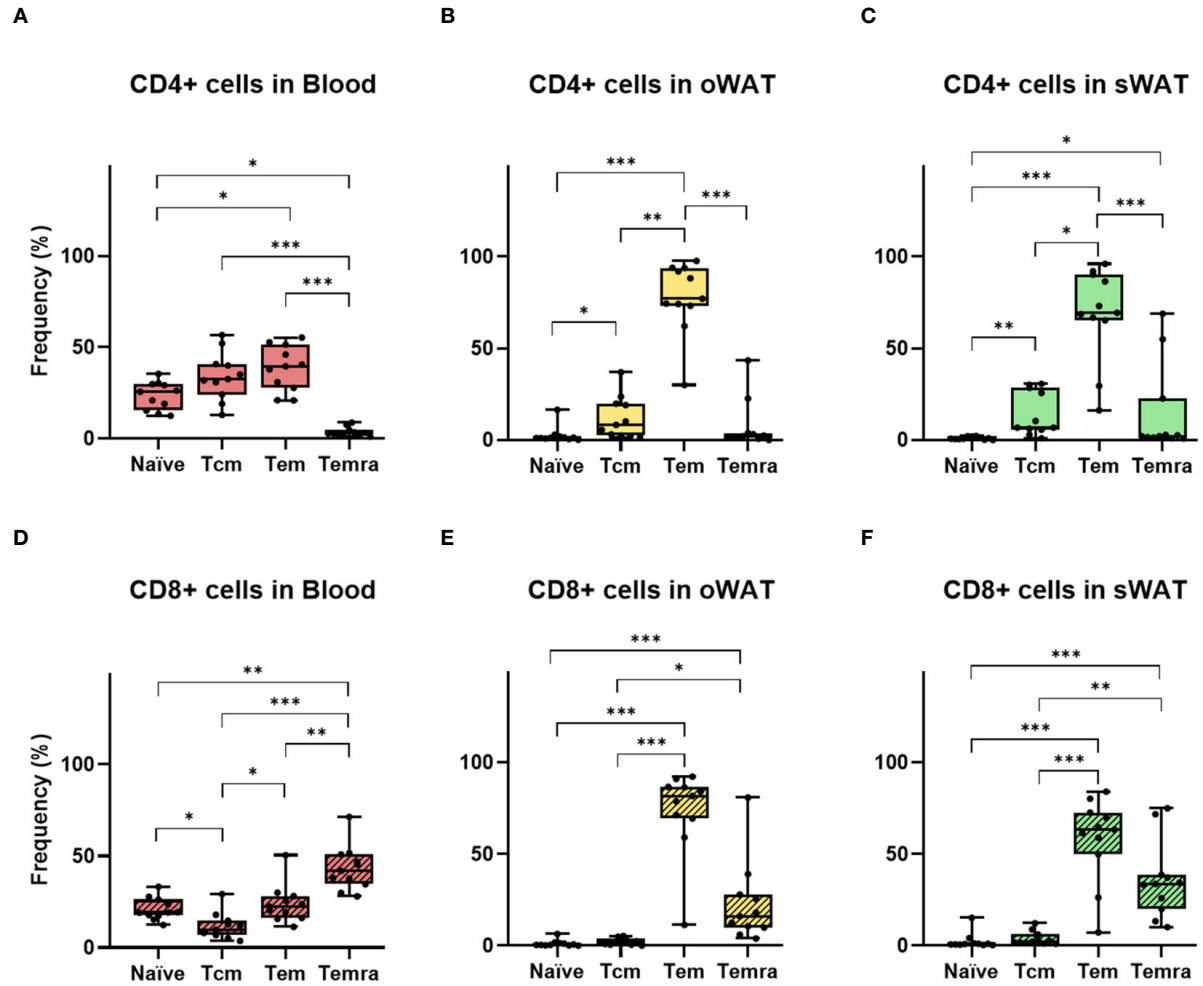
Differences between the number of naïve and memory T cells in CD4^+^ and CD8^+^ independently. **(A)** CD4+ subsets (T central memory, T effector memory, T effector memory re-expressing CD45RA) in blood. **(B)** CD4+ subsets in oWAT. **(C)** CD4+ subsets in subcutaneous sWAT. **(D)** CD8+ subsets in blood. **(E)** CD8+ subsets in oWAT. **(F)** CD8+ subsets in sWAT. *: p ≤ 0.05, **: p ≤ 0.01, ***: p ≤ 0.001.

**Supplementary Figure 2A** shows the SSC *versus* FSC plot of oWAT SVF and the gating strategy to identify CD4+ and CD8+ populations by Panel 2. Noteworthy, blood and tissue samples displayed different flow cytometry profiles (**Supplementary Figures 2B, C**). Such indicated that contamination with peripheral blood in tissue samples was negligible. One of the main differences shown by panel 1 was in the naïve population, which was abundant in blood but marginal in tissue (22.2% blood, 1.9% oWAT, 1.7% sWAT, p<0.001, **Figure 4**). Furthermore, the proportion of T cells positive for tissue-resident markers was different in blood and WAT, highlighting the fact that almost all T cells in tissue have a memory phenotype. As expected, CD103 and CD49a were nearly absent in blood (**Supplementary Figures 2C, D**, **3**) and only a small fraction of T cells was positive for CD69 (CD4: 2.7%, CD8: 4.4%, **Supplementary Figure 2C**). In contrast, most adipose tissue T lymphocytes were positive for CD69 (CD4-oWAT: 86.8%, CD8-oWAT: 93.3%, CD4-sWAT: 72.5%, CD8-sWAT: 81.7%, **Supplementary Figure 2C**).

**Figure 5:**
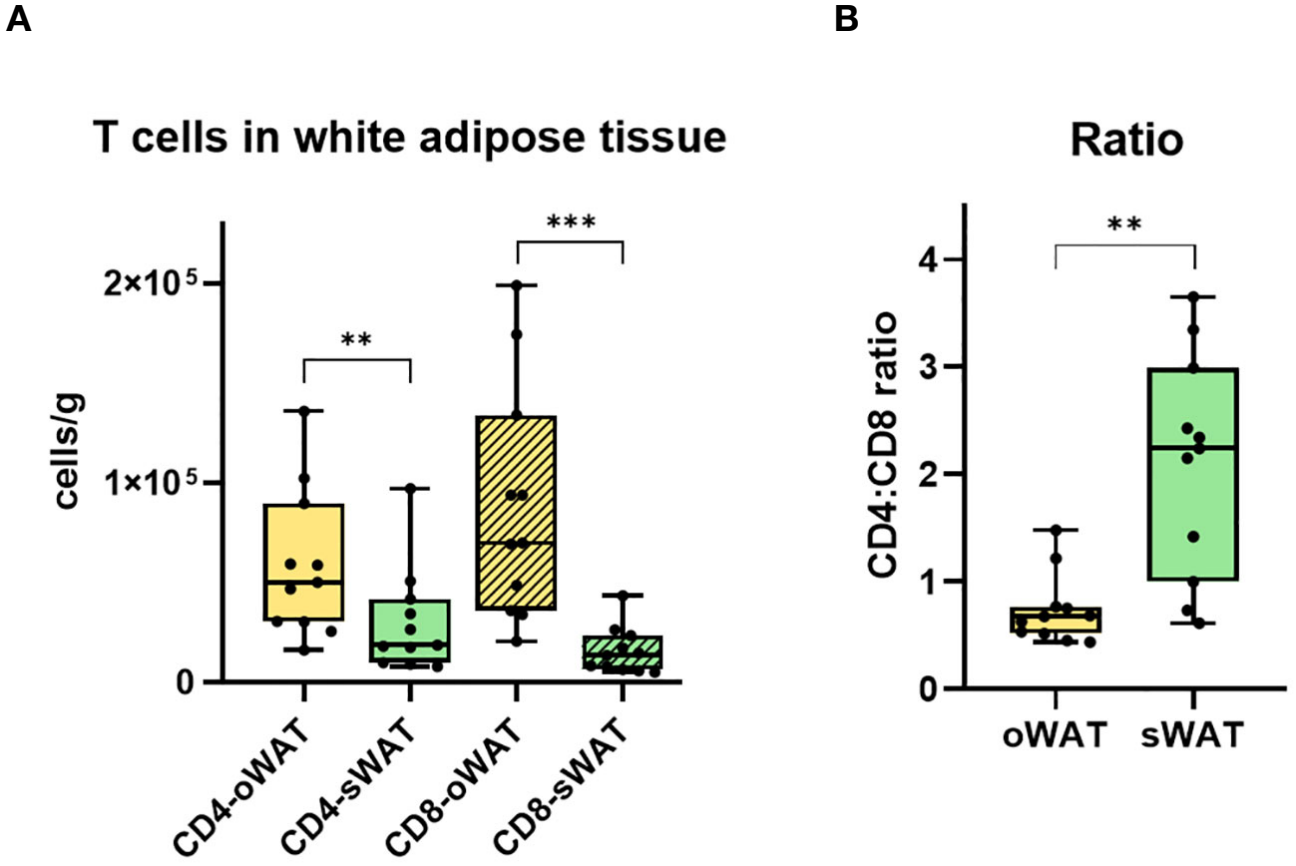
Differences in CD4+ and CD8+ T cells in adipose tissue. **(A)** Total number of CD4+ and CD8+ T lymphocytes per gram of oWAT and sWAT. **(B)** Ratio CD4:CD8 in omental and subcutaneous adipose tissue. **: p ≤ 0.01, ***: p ≤ 0.001.

There were significantly more CD4+ and CD8+ T lymphocytes in oWAT than in sWAT (**Figure 5A**). This agrees with previous reports showing that FALCs are more abundant in oWAT than in sWAT (14, 15). Moreover, the CD4+:CD8+ ratio differed significantly between both types of adipose tissue (**Figure 5B**). In oWAT CD8+ cells dominated the T cell pool, while in sWAT CD4+ cells did so. Therefore, the increase of CD8+ T cells in oWAT was the main driving force behind the differences in the number of T cells between oWAT and sWAT.

In adipose tissue samples, naïve T cells were almost absent, which agrees with previous reports (23) (**Figures 4B, C, E, F**). In both adipose tissues, the most abundant CD4+ subset was Tem, followed by Tcm and Temra (**Figures 4B, C**). In the CD8+ subpopulation Tem was the larger subset in both tissues as well, followed by Temra and Tcm (**Figures 4E, F**). Furthermore, in blood samples, there were around 22% of naïve T cells in both CD4+ and CD8+ subpopulations (**Figures 4A, D**). It is worth noting that the number of naïve T cells can vary within individual’s age since their production by the thymus decreases with senescence (24). Remarkably, in the CD8+ subpopulation of blood, Temra was the most abundant subset (**Figure 4D**) whereas it was almost absent in the CD4+ subset (**Figure 4A**). This was consistent with previous results, which described circulant Temra CD8+ as a major subset that increases with age (25).

**Figure 6:**
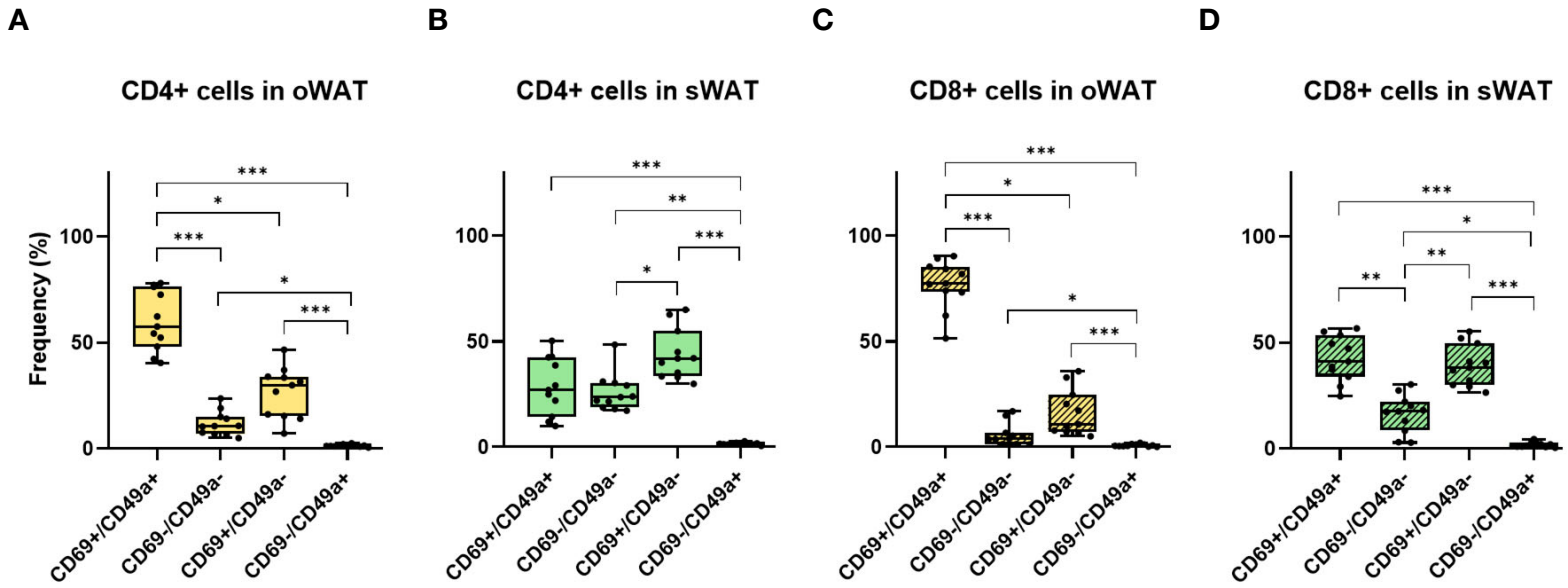
Differences between CD69 and CD49a profile for CD4+ and CD8+ in oWAT and sWAT. **(A)** CD4+ subsets in oWAT. **(B)** CD4+ subsets in sWAT. **(C)** CD8+ subsets in oWAT. **(D)** CD8+ subsets in sWAT. *: p ≤ 0.05, **: p ≤ 0.01, ***: p ≤ 0.001.

In Trm cells, CD69 is usually co-expressed with CD49a and/or CD103. The percentage of CD103-positive T cells was minimal in adipose tissue (**Supplementary Figures 2D**, **3**) whereas CD49a showed high levels, especially in oWAT (**Supplementary Figure 2C**). From CD69 and CD49a expression patterns, we extract two conclusions. Firstly, virtually all CD49a-positive T cells co-expressed CD69. Secondly, the double positive population (CD69+/CD49a+) was the most abundant in oWAT (particularly in the CD8+ subpopulation) (**Figures 6A, C**) while there was not a predominant population in sWAT (**Figures 6B, D**).

## 3 Discussion

### 3.1 Differences in human and mice adipose tissue

A growing body of evidence supports the immunological function of WAT (8). Nevertheless, most of the data are derived from murine models and they significantly differ from humans in their anatomy and physiopathology of adipose tissue (26). Humans have two main visceral fat depots in the intraperitoneal cavity (omental and mesenteric). Conversely, mice have a mesenteric and a well-developed gonadal depot but lack the omental depot, only visible in obese animals (27). The principal fat depots with the ability of expansion are subcutaneous and certain visceral depots. Concerning visceral expansion, rodents expand their gonadal depot, whereas humans mainly expand the omental fat depots, and both tissues differ notably. While murine gonadal fat drains to the systemic circulation, human omental adipose tissue drains to the portal circulation. Moreover, the presence of immune cells and FALCs is higher in human oWAT (14). This strategic location gives oWAT the opportunity of filtering a more diverse antigen repertoire. In both species an excessive expansion of intraperitoneal fat depots is associated with obesity-related pathologies (28). On the other hand, mesenteric depots have been much less studied, unlike omental and gonadal depots, due to the small quantity of this tissue in mice and the difficulty of taking biopsies in humans.

Our work is pioneering in studying the immunological component of oWAT in humans. Nevertheless, our data must be interpreted in the context of morbid obesity, where the excessive expansion of body fat depots changes the composition and phenotype of adipose tissue leukocytes. Obesity increases the infiltration of neutrophils and reduces the number of resident mast cells (9, 10). Furthermore, obesity induces a pro-inflammatory phenotypic switching in macrophages and disrupts the function of mast cells (11, 29). This state of low-level chronic inflammation, together with other factors (such as adipocyte hypertrophy, fibrosis, or tissue hypoxia) impairs the normal function of adipose tissue, promoting obesity-associated pathologies.

### 3.2 TCR-antigen prediction

Due to the low accuracy of current TCR antigen specificity prediction algorithms (30), the best TCR annotation strategy is cross-referencing with a database such as VDJdb. At the time of analysis, VDJdb listed 34685 TCR-antigen-HLA trios, which allowed us to annotate only 0.42% of our TCRs. The main limitation of these databases is that they contain antigen-specificity information for a negligible fraction of the possible TCRs that can be generated by the somatic recombination process. Furthermore, viral antigens are currently overrepresented, which leaves many non-viral epitopes without experimentally validated cognate TCR (30).

Although we have performed high-resolution HLA genotyping of our cohort, we did not set HLA matching as a strict filter to rule out antigen specificity annotations. The genetics of HLA is an intricate system (20, 21): hyper-polymorphic, multigenic and codominant. Moreover, it has been shown a proinflammatory environment may change the collection of peptides presented by HLA class I and II molecules (31, 32). Furthermore, the interaction between TCR and the antigen-HLA complex shows a wide spectrum of engagement, from high-affinity and high-specific interactions to low-affinity and promiscuous interactions.

Despite all the aforementioned limitations, our *in silico* antigen specificity analysis predicted the presence of CMV-specific T lymphocytes infiltrating adipose tissue and this was successfully validated in subsequent functional assays. This analysis would greatly benefit from future updates of VDJdb, providing a more comprehensive TCR annotation to decipher the full landscape of antigen specificity of adipose tissue-infiltrating T lymphocytes.

### 3.3 Reactivity against CMV antigens by ELIspot assay

The ELIspot assay reveals certain particularities in oWAT, concerning the composition of the T cells reservoir. Firstly, the number of spots was significantly more elevated in oWAT than in blood and sWAT, showing a clear enrichment in CMV-reactive T cells. One previous study performed in human samples found relevant frequencies of T cells against CMV-antigens in blood, bone marrow, and lymphoid nodes but negligible in intestinal mucosa (19). Nevertheless, this study did not analyze adipose tissue samples. Secondly, although we lack specific experiments to confirm that T cells reacting in the ELISpot assay are Trm-like, there is a significant observation to consider. More than 95% of oWAT T cells show characteristics of memory T cells by flow cytometry. Additionally, there is negligible contamination with peripheral blood in tissues, so very few naïve T cells appear in tissue. Thirdly, the spot size was significantly larger in oWAT, which can be conditioned by cell phenotype and/or the TCR binding affinity.

In our cohort, some patients showed discrepancies between the antigen-specificity prediction and the ELISpot assays. Bioinformatics analyses predicted the presence of CMV-specific TCRs in blood but not in WAT (considering a 0.0001 frequency threshold). However, all of them were positive in the ELISpot assay in both blood and WAT. There could be several reasons (methodological and biological) for this discrepancy. On the one hand, we could have lost some TCR clonotypes due to sampling bias and/or quality controls and filters of subsequent data processing. On the other hand, the clonotype composition significantly differs between blood and oWAT. This phenomenon, together with the fact that most of VDJdb database annotations come from experiments with PBMCs, may cause the loss of relevant information.

### 3.4 Characteristics of T resident memory cells in adipose tissue

In a previous article, we reported that significant weight loss in subjects with morbid obesity did not alter either the proportion of adipose tissue T lymphocytes (into the ensemble of non-adipose cells) or the CD4+:CD8+ ratio (9). We do not have any data about the status of the adipose-tissue Trm reservoir in human lean subjects. Nevertheless, it is reasonable to think that the visceral fat depots of lean individuals act as a relevant reservoir of T cell memory as well. Further studies are needed to elucidate how obesity impacts the normal function of the adipose-tissue Trm reservoir. Additionally, Trm population may affect the development of obesity-related pathologies. In this way, a study in a murine model suggested that the infection of mCMV in its acute phase might impair the glucose metabolism (4). Moreover, other relevant issues remain unsolved, such as the immunological differences between human intraperitoneal fat depots (especially, omental and mesenteric depots), or their relationship with mucosa-associated immunity. What is established, and important to highlight, about the intraperitoneal fat depots examined in this study is that oWAT contains a higher abundance of FALCs and immune cells in comparison with sWAT.

One remarkable finding is the elevated proportion of CD8+ T cells in oWAT. The relative abundance of CD4+ and CD8+ T lymphocytes is controlled by different factors and is biologically relevant. The thymus produces more CD4+ than CD8+ lymphocytes (33) but the ratio between CD4+ and CD8+ T lymphocytes greatly varies between peripheral tissues (34). In general, CD4+ dominate the T cell pool in almost the entire body except in the intestine, where CD4+ and CD8+ T lymphocytes are in similar amounts (34). Nevertheless, both subpopulations expand with important differences under immunological stimulation (like a viral infection). In the case of CD4+ T cells, expansion is coupled with IFN-γ-mediated apoptosis. Thus, the number of CD4+ T cells does not significantly change after the stimulation. Oppositely, CD8+ T cells do not have an apoptosis mechanism coupled with expansion. Therefore, CD8+ T cells significantly increase after stimulation (35). When the immunological stimulation is low, the expansion of CD8+ T cells is mainly driven by the TCR recognition of the cognate epitope in an HLA class I molecule. When the immunological stimulation is high, CD8+ T cells can proliferate in a bystander way (TCR independent). This type of activation is characteristic of Tem and is quite infrequent in other subsets. In short, the CD8+ domination of the T cell pool in peripheral tissues is a marker of immunological stimulation.

CD69, a type II C-lectin receptor, is a classical early marker of lymphocyte activation, and also a marker of tissue retention (36). CD69 is widely expressed in Trm lymphocytes (37, 38) whereas the expression in circulant T cells, which is undetectable at steady-state, may be increased in diverse pathological contexts (36). CD69 is usually co-expressed in skin and mucosal tissues with other Trm markers, such as integrins CD49a and CD103. The expression pattern of Trm markers determines different phenotypes. For instance, CD49a seems to be essential for long-term persistence of Trm lymphocytes, in part by generating survival signals through the interaction with collagen IV (39, 40). Moreover, CD49a expression is associated with increased T-cells motility (41). Importantly, the recurrence of viral infections increases the proportion of CD49a+ cells in Trm lymphocytes (42–44). These data are in coherence with the prominence of the CD49a+ subset in adipose tissue T lymphocytes, particularly in oWAT CD8+ cells (42). In contrast, the expression of CD103 is almost absent in adipose tissue. This marker, which is important for epithelial retention of memory T cells (38, 45), does not appear relevant in the adipose tissue context. In our study, the expression pattern of CD69 and CD49a defined three main populations (CD69+/CD49a+, CD69+/CD49a- and CD69-/CD49a+) with presumably different roles. These results open an interesting research field. Future studies should include new Trm markers and characterize the role of different T cell populations in adipose tissue depots.

### 3.5 Adaptive immune response in the context of viral infection

The adaptive immune response against pathogens always begins with a rapid expansion of the clones reactive to pathogen antigens. Once the infection is controlled, this T cell population is contracted to avoid the deleterious effects of excessive activation of these cells. At this point, three different scenarios can occur: 1) if the pathogen is completely controlled and cleared, a memory population will remain stable over time to protect against reinfection; 2) if the pathogen is not completely controlled, it establishes a chronic infection, like HIV or hepatitis C virus (HCV), where the pathogen antigens are always present and T cells are continuously challenged. This causes the accumulation of memory cells over time that become exhausted. However, this term is being revised lately as phenotypical adaptation in the context of chronic inflammation by some authors (46); 3) if the pathogen is controlled (so it is undetectable) but it is not completely cleared, it establishes a latent infection. These latent pathogens are dormant during the lysogenic part of their cycle and thus are not detected by the immune system. However, they can periodically be activated and enter the lytic part of their cycle to reactivate the infection (47).

Oppositely to chronic viruses, the presence of antigens from latent viruses is not constant. Thus, there is no accumulation of exhausted T cells. However, the persistent reactivation of the infection triggers the accumulation of CD8+ Tem cells in a process called memory inflation. Besides, this memory T cell population behaves differently than normal memory T cell populations. Under normal conditions, Tem cells have a limited expansion capacity, and this pool relies on the proliferation and differentiation of Tcm cells (long-lived classical memory). Nevertheless, in latent infections Tem cells substantially increase their proliferation rate and maintain their pool without the support of Tcm cells (48). Such generates a new long-lived non-classical memory population in the Tem pool (49). Interestingly, the latent infection promotes changes not only in the phenotype and dynamics of the memory T cell population but also in the TCR repertoire.

Unlike antibodies, TCRs lack a mechanism for affinity maturation. Therefore, under normal conditions, very few T cells would be able to bind the HLA-peptide complex with enough affinity to generate a response and a memory population. Consequently, few mutations in the virus would be enough to escape the T cell response. Fortunately, the coreceptor molecules CD4 and CD8 solve this problem by increasing the stability of the TCR-peptide-HLA complex by binding conserved regions in the HLA. Such allows TCRs with moderate affinity to enter the memory compartment increasing its diversity and preventing immune escape through antigen mutation (50). This mechanism works very efficiently when the antigen load is high, like during the acute phase of the infection: TCRs with high affinity are saturated and low-affinity TCRs are able to bind the remaining antigens. However, in the latent phase, the antigen load is small, and the high-affinity TCRs outcompete the low-affinity TCRs. Therefore, after several lytic cycles, the response becomes very oligoclonal and directed to the most immunodominant antigens. For instance, the phosphoprotein pp65 and the immediate-early protein 1 (IE1) of CMV (49). This phenomenon may increase its relevance with age. This progressively more skewed response allows the reinfection with a genetically different variant of the latent virus in a process called superinfection (51). Importantly, this phenomenon has hampered the efforts to develop a vaccine against CMV or EBV (52). On top of that, CMV and EBV have a vast arsenal of immunoevasins to escape the immune response.

In conclusion, our results showed that adipose tissue, mainly oWAT, hosts a complete immune response against latent CMV infection, and presumable to other viruses (including EBV and influenza) or other pathogens. Thus, our study suggests that the immunological function is more robust in oWAT than sWAT. We found CMV-reactive T lymphocytes, inflation of the CD8+ population, and the skewed TCR repertoire against immunodominant antigens presented by HLA class I alleles. This strongly suggests that oWAT is a relevant immunological organ for antiviral responses in humans. Although our study has specifically examined oWAT and sWAT, other types of adipose tissue (even beyond the intraperitoneal cavity) may also play a relevant immunological role.

## 4 Materials and methods

### 4.1 Patient cohort

This study includes 11 morbidly obese patients who underwent laparoscopic bariatric surgery (gastric bypass or gastric sleeve) at San Cecilio University Hospital (Granada, Spain). The ethics committee of the hospital (Andalucia’s Biomedical Research Ethics Committee of Granada) approved the study, and all participants signed written informed consent. Patients were excluded from the cohort if they presented any autoimmune disorder. All biochemical analyses were carried out within 24 hours by routine methods at the Clinical Analysis Laboratory of San Cecilio University Hospital (Granada, Spain). HLA genotyping, CMV and EBV serology were carried out at the Clinical Analyses and Immunology Unit of Virgen de las Nieves University Hospital (Granada, Spain). HLA genotyping was performed through PCR-SSO (Specific Sequence Oligonucleotides) with LABType kits from One Lambda (Los Angeles, CA, USA). PCR-SSO uses probes that hybridize to the most polymorphic regions of each *locus*. On the other hand, CMV and EBV serology were performed through CLIA (ChemiLuminescent ImmunoAssay), an ultrasensitive ELISA, with Alinity i CMV IgG and IgM, and EBV VCA IgM and EBNA-1 IgG kits by Abbott (Chicago, IL, USA). In the case of CMV, no specific antigen is used, but a total lysate of CMV from AD169 strain.

### 4.2 Sample processing

Blood samples were collected before surgery and after 10 h fasting. Liver (around 10 mg) and adipose tissue biopsies (around 3 g) were obtained at the moment of surgery and kept in PBS at 4 °C until they arrived at the laboratory. oWAT biopsies were obtained from the greater omentum close to the stomach. sWAT tissue biopsies were obtained near the surgical incision.

Liver biopsies were entirely devoted to TCR analysis. Therefore, liver biopsies were preserved in 500 μl of lysis buffer (Qiagen, Hilden, Germany) at -20°C until total RNA extraction. Around 300 mg of the biopsies from each adipose tissue were preserved in 500 μl of lysis buffer (Qiagen) at -20°C for RNA extraction. The rest of each adipose tissue biopsy (around 2 g) was examined to remove small lymph nodes and blood vessels that were eventually present in the tissue. After that, they were digested in 10 ml of RPMI 1640 medium supplemented with 2 mg/ml type I collagenase (Sigma, St Louis, MO, USA), 1 mg/ml hyaluronidase (Sigma) and 5 mM CaCl2, for 2h, at 37°C.

The digestion was diluted with PBS and filtered through a 1 mm sieve. Later, it was centrifuged at 900 x g for 10 minutes and the pellet was resuspended in PBS, then filtered through a 100 μm filter, and centrifuged again at 900 x g for 10 minutes. The resulting pellet contained the SVF, and it was split for flow cytometry and ELISpot.

For flow cytometry, a quarter of the SVF was resuspended in 200μl of antibody staining buffer (PBS, 2% fetal bovine serum, 0.09% albumin, and 0.05% sodium azide) and mixed with an internal standard (CountBright Absolute Counting Beads). The internal standard is a suspension of a known number of autofluorescent beads with wide emission spectra. The internal control has different size and complexity values from any cell population, which enables the differentiation between them. For ELISpot, the rest of the SVF was resuspended in 1 ml of RPMI 1640 medium to isolate the mononuclear fraction from the SVF by ficoll gradient.

Likewise, PBMCs from 6 ml of blood samples diluted 1:1 with PBS were also isolated by ficoll gradient. The interphase obtained was washed with PBS and pellet was later preserved in 500 μl of lysis buffer at -20°C for RNA extraction.

### 4.3 RNA extraction and retrotranscription

Total RNA extraction from isolated peripheral blood lymphocytes and tissues (oWAT, sWAT, and liver) was carried out using standard silica-membrane columns from RNeasy Mini Kit by Qiagen. A minimum of 20ng/µl of RNA was retrotranscribed to cDNA using the iScript™ cDNA Synthesis Kit by BioRad (Hercules, CA, USA).

### 4.4 TCRβ library preparation and next-generation sequencing (NGS)

TCRβ repertoire sequencing was performed in the Ion S5 platform using Ion-530 chips and its extended modality, which can obtain reads up to 600pb. The coverage was around 140000 sequences per sample. During the preparation of NGS libraries, one of the main sources of bias is the ligation reaction, which is usually employed to join adaptors (53). For that reason, our innovative methodology prepared long-read TCRβ barcoded libraries without any ligation step. Moreover, as opposed to most current strategies based on short reads centered in the CDR3 (sequencing only the V segment end region), our long-read method covered the entire V segment. This improved the identification within highly conserved V families, particularly in V5 and V6 families. Reads also included D and J segments, a small part of C segment, the CDR-3 loop (functional part of the hypervariable region) and, CDR-1 and CDR-2 loops (provided by each V segment).

The methodology for TCRβ library preparation is fully detailed Supplementary Protocol 1. It involved an enrichment multiplex PCR where all TCRs were amplified simultaneously with a collection of primers capable of hybridizing with every V segment. It was followed by the “fishing” of the molecules of interest by magnetic particles. After that, the sequences of the adaptors for Ion S5 (A and P1) were added through the extension on the DNA strands covalently bound to the magnetic particles. This step was critical since it avoided ligation bias. Lastly, there was a final PCR to release the DNA from the microparticles and the purification of its product.

### 4.5 TCR repertoire analysis

Read length distribution and sequencing quality were assessed with FastQC and MultiQC (54) software. BAM files were filtered with SAMtools (55) to select reads between 400-500 nucleotides long, as it was the expected length range of our TCRβ amplification method. Demultiplexing by sample barcode identification, tetramer (CGAT) and C primer sequence integrity were ascertained with R package Biostrings (v2.50.2) (56). Reads that did not meet any of these criteria were discarded from the analysis. Identification of V and J gene segments and CDR3 sequence was performed with RTCR v0.5.1 (57) with default parameters.

Immune repertoire analyses were performed with the immunarch R package (v0.6.5). A unique clonotype was defined by its V and J gene segments and its CDR3 amino acid sequence. Proportional downsampling and bootstrapping with 100 replicates were performed in the diversity and overlap analyses (except for Venn diagrams) to account for differences in read counts between samples. Productive clonality was calculated as the square root of Simpson’s diversity index of productive clonotypes.

### 4.6 Antigen specificity prediction

Antigen specificity prediction was performed following the guidelines by Pogorelyy and Shugay (2019) (58). First, the VDJdb database (59) was downloaded in June 2021 and all the TCRβ CDR3 sequences were clustered with our repertoires with GIANA (Geometric Isometry-based TCR AligNment Algorithm) (60) in query mode, considering V gene segment and CDR3 amino acid sequence. All query TCRs that clustered with a TCR present in the VDJdb database were annotated with the antigen specificity information provided in the database. Only antigens whose cognate TCRs represented an aggregated frequency in the repertoire higher than 0.0001 (medium-sized, large-sized and hyperexpanded clones) were included in the analysis to control for unspecific annotations lower in frequency that may not reflect a true T-cell response.

### 4.7 ELISpot assays

ELISpot was performed with ELISpot Path PRO: Human IFN-γ (CMV) (Mabtech, Nacka Strand, Sweden). The CMV peptide included in the ELISpot assay consists of 42 peptides from CMV that induces the secretion of IFN-γ, from human CD4 and CD8 T cells. The antigens presented in the pool are pp50, pp65, IE1, IE2 and envelope glycoprotein B. Of the 42 peptides, 28 are HLA-I restricted (covering HLA-A1, HLA-A2, HLA-A3, HLA-A11, HLA-A23, HLA-A24, HLA-A26, HLA-A30, HLA-B7, HLA-B8, HLA-B18, HLA-B27, HLA-B35, HLA-B40/60, HLA-B41, HLA-B44, HLA-B57/58, HLA-C7) and 14 are HLA-II restricted (covering HLA-DRB1*01, 03, 04, 05, 07, 08, 11, 15, 20, 24, 53, HLA-DPw2, HLA-DQB, HLA-DP3*14, 20). Beforehand, we ensured that every donor in our cohort carry at least one HLA restriction allele, so that the pool covers all the donors.

The ELISpot plate, precoated with 1-D1K monoclonal antibody, was washed 4 times with sterile PBS and incubated with RPMI + 10% FBS medium for 2 hours. According to the manufacturer’s instructions, duplicates (when possible) of 250000 cells from the mononuclear fraction of SVF and PBMCs were seeded in each well. Cells were resuspended in 100 μl of RPMI + 10% FBS medium with 2μg/ml of CMV peptide pool for human CD4 and CD8 T cells. Positive control cells were resuspended in media with 1:1000 anti-CD3. One negative control was also included. Positive and negative controls were performed only with PBMCs due to a lack of cells in oWAT and sWAT. Plates were incubated for 16 - 24 hours at 37°C in a 5% CO2 incubator. After 5 PBS washing steps, 100μl diluted 7-B6-1-ALP conjugate (1μg/ml) was added per well and incubated for 2 hours at room temperature. After another 5 times of PBS washes, 100μl of filtered BCIP/NBT- plus solution was added per well and left until spots emerged. To stop the color reaction, plates were washed with tap water. After letting the plate dry, photographs were taken with a FujiFilm X-T4 camera and a Canon macro lens of 100 mm. Spots were then counted, and their area was measured with ImageJ.

### 4.8 Flow cytometry

The SVF or fresh blood of the 11 donors was labelled with 2 ml of controls or fluorophore-conjugated antibodies at room temperature for 20 minutes. Later, cells were fixed, and the erythrocytes were lysed with 1 ml of BD FACS Lysing Solution for 30 minutes. After that, samples were centrifuged for 10 minutes at 3500 x g, and pellets were resuspended in 200 µl of PBS. Then, samples were stored at 4°C until the next day.

Flow cytometry was performed using BD FACS ARIA IIIu equipment, and data were generated with a logarithmic scale acquisition. The internal standard was used to calculate the number of cells per mg of tissue. Two panels with different fluorescent-conjugated antibodies were used. Panel 1: anti-CD3 BB515 (clone HIT3A, BD Biosciences), anti-CD4 APC (clone RPA-T4, BD Biosciences), anti-CD8 BV711 (clone SK1, BioLegend), anti-CCR7 PE-Cy7 (clone G043H7, BioLegend) and anti-CD45RA BV421 (clone HI100, BioLegend). Panel 2: anti-CD3 BV510 (clone OKT3, BioLegend), anti-CD4 FITC (clone RPA-T4, BD Biosciences), anti-CD8 BV711 (clone SK1, BioLegend), anti-CD69 PE-Cy7 (clone FN50, BD Biosciences), anti-CD49a APC (clone TS2/7, BioLegend) and anti-CD103 PE (clone Ber-ACT8, BioLegend).

### 4.9 Statistical tests

Unless otherwise specified, all statistical analyses were performed in R (v3.6.1). The normality of the data was assessed with the Shapiro-Wilk test and from there it was decided to conduct non-parametric tests. Kruskal-Wallis test was chosen to compare groups and *post-hoc* pairwise comparisons were performed with the two-sided Wilcoxon test. Graphics on **Figures 3**–**6** were performed with GraphPad 8 (La Jolla, CA, USA).

## Data availability statement

The datasets presented in this study can be found in online repositories. The names of the repository/repositories and accession number(s) can be found below: BioProject accession: PRJNA980167.

## Ethics statement

The studies involving humans were approved by Andalucia’s Biomedical Research Ethics Committee of Granada. The studies were conducted in accordance with the local legislation and institutional requirements. The participants provided their written informed consent to participate in this study.

## Author contributions

AR: Conceptualization, Formal Analysis, Investigation, Methodology, Validation, Visualization, Writing – original draft, Writing – review & editing. MB: Conceptualization, Data curation, Formal Analysis, Methodology, Validation, Visualization, Writing – original draft, Writing – review & editing. DL: Investigation, Writing – original draft, Writing – review & editing. JG: Resources, Writing – review & editing. FT: Resources, Writing – review & editing. DP: Investigation, Software, Writing – review & editing. SM: Software, Visualization, Writing – review & editing. IR: Resources, Visualization, Writing – review & editing. JP: Investigation, Writing – review & editing. JV: Investigation, Writing – review & editing. ML: Investigation, Writing – review & editing. FG: Supervision, Writing – review & editing. CC: Funding acquisition, Supervision, Writing – review & editing. JL: Conceptualization, Funding acquisition, Project administration, Supervision, Writing – review & editing. ÁC: Conceptualization, Funding acquisition, Project administration, Supervision, Writing – original draft, Writing – review & editing.

## References

[1] Fowler K Mucha J Neumann M Lewandowski W Kaczanowska M Grys M. A systematic literature review of the global seroprevalence of cytomegalovirus: possible implications for treatment, screening, and vaccine development. BMC Public Health (2022) 22(1):1659. doi: 10.1186/s12889-022-13971-7 36050659 PMC9435408

[2] Holtappels R Pahl-Seibert MF Thomas D Reddehase MJ. Enrichment of immediate-early 1 (m123/pp89) peptide-specific CD8 T cells in a pulmonary CD62L(lo) memory-effector cell pool during latent murine cytomegalovirus infection of the lungs. J Virol (2000) 74(24):11495–503. doi: 10.1128/JVI.74.24.11495-11503.2000 PMC112429 11090146

[3] Emery VC. Investigation of CMV disease in immunocompromised patients. J Clin Pathol (2001) 54(2):84–8. doi: 10.1136/jcp.54.2.84 PMC1731357 11215290

[4] Contreras NA Sitnik KM Jeftic I Coplen CP Čičin-Šain L Nikolich-Žugich J. Life-long control of cytomegalovirus (CMV) by T resident memory cells in the adipose tissue results in inflammation and hyperglycemia. PLoS Pathogens (2019) 15(6):e1007890. doi: 10.1371/journal.ppat.1007890 31220189 PMC6605679

[5] Wanjalla CN McDonnell WJ Ram R Chopra A Gangula R Leary S. Single-cell analysis shows that adipose tissue of persons with both HIV and diabetes is enriched for clonal, cytotoxic, and CMV-specific CD4+ T cells. Cell Rep Med (2021) 2(2):100205. doi: 10.1016/j.xcrm.2021.100205 33665640 PMC7897802

[6] Scheja L Heeren J. The endocrine function of adipose tissues in health and cardiometabolic disease. Nat Rev Endocrinol (2019) 15(9):507–24. doi: 10.1038/s41574-019-0230-6 31296970

[7] Wu J Cohen P Spiegelman BM. Adaptive thermogenesis in adipocytes: Is beige the new brown? Genes Dev (2013) 27(3):234–50. doi: 10.1101/gad.211649.112 PMC3576510 23388824

[8] Trim WV Lynch L. Immune and non-immune functions of adipose tissue leukocytes. Nat Rev Immunol (2022) 22(6):371–86. doi: 10.1038/s41577-021-00635-7 34741167

[9] García-Rubio J León J Redruello-Romero A Pavón E Cozar A Tamayo F. Cytometric analysis of adipose tissue reveals increments of adipocyte progenitor cells after weight loss induced by bariatric surgery. Sci Rep (2018) 8(1):15203. doi: 10.1038/s41598-018-33488-7 30315279 PMC6185966

[10] Lopez-Perez D Redruello-Romero A Garcia-Rubio J Arana C Garcia-Escudero LA Tamayo F. In patients with obesity, the number of adipose tissue mast cells is significantly lower in subjects with type 2 diabetes. Front Immunol (2021) 12:664576. doi: 10.3389/fimmu.2021.664576 34093556 PMC8177010

[11] Lopez-Perez D Redruello-Romero A Garcia-Rubio J Arana C Garcia-Escudero LA Tamayo F. In obese patients with type 2 diabetes, mast cells in Omental adipose tissue decrease the surface expression of CD45, CD117, CD203c, and fcϵRI. Front Endocrinol (Lausanne) (2022) 13:818388. doi: 10.3389/fendo.2022.818388 35370964 PMC8965342

[12] Unamuno X Gómez-Ambrosi J Rodríguez A Becerril S Frühbeck G Catalán V. Adipokine dysregulation and adipose tissue inflammation in human obesity. Eur J Clin Invest (2018) 48(9):e12997. doi: 10.1111/eci.12997 29995306

[13] Zhang Lj Guerrero-Juarez CF Hata T Bapat SP Ramos R Plikus MV. Dermal adipocytes protect against invasive Staphylococcus aureus skin infection. Science (2015) 347(6217):67–71. doi: 10.1126/science.1260972 25554785 PMC4318537

[14] Bénézech C Luu NT Walker JA Kruglov AA Loo Y Nakamura K. Inflammation-induced formation of fat-associated lymphoid clusters. Nat Immunol (2015) 16(8):819–28. doi: 10.1038/ni.3215 PMC4512620 26147686

[15] Moro K Yamada T Tanabe M Takeuchi T Ikawa T Kawamoto H. Innate production of TH2 cytokines by adipose tissue-associated c-Kit+Sca-1+ lymphoid cells. Nature (2010) 463(7280):540–4. doi: 10.1038/nature08636 20023630

[16] Meza-Perez S Randall TD. Immunological functions of the omentum. Trends Immunol (2017) 38(7):526–36. doi: 10.1016/j.it.2017.03.002 PMC5812451 28579319

[17] Han SJ Zaretsky AG Andrade-Oliveira V Collins N Dzutsev A Shaik J. The white adipose tissue is a reservoir for memory T cells that promotes protective memory responses to infection. Immunity (2017) 47(6):1154–68. doi: 10.1016/j.immuni.2017.11.009 PMC5773068 29221731

[18] Damouche A Lazure T Avettand-Fènoël V Huot N Dejucq-Rainsford N Satie AP. Adipose tissue is a neglected viral reservoir and an inflammatory site during chronic HIV and SIV infection. PLoS Pathogens (2015) 11(9):e1005153. doi: 10.1371/journal.ppat.1005153 26402858 PMC4581628

[19] Gordon CL Miron M Thome JJC Matsuoka N Weiner J Rak MA. Tissue reservoirs of antiviral T cell immunity in persistent human CMV infection. J Exp Med (2017) 214(3):651–67. doi: 10.1084/jem.20160758 PMC5339671 28130404

[20] Meyer D C Aguiar VR Bitarello BD C. Brandt DY Nunes K. A genomic perspective on HLA evolution. Immunogenetics (2018) 70(1):5–27. doi: 10.1007/s00251-017-1017-3 28687858 PMC5748415

[21] Rao X Hoof I Fontaine Costa AICA van Baarle D Keşmir C. HLA class I allele promiscuity revisited. Immunogenetics (2011) 63(11):691–701. doi: 10.1007/s00251-011-0552-6 21695550 PMC3190086

[22] Mandelcorn-Monson RL Shear NH Yau E Sambhara S Barber BH Spaner D. Cytotoxic T lymphocyte reactivity to gp100, melanA/MART-1, and tyrosinase, in HLA-A2-positive vitiligo patients. J Invest Dermatol (2003) 121(3):550–6. doi: 10.1046/j.1523-1747.2003.12413.x 12925214

[23] Farber DL Yudanin NA Restifo NP. Human memory T cells: generation, compartmentalization and homeostasis. Nat Rev Immunol (2014) 14(1):24–35. doi: 10.1038/nri3567 24336101 PMC4032067

[24] Palmer D. The effect of age on thymic function. Front Immunol (2013) 4:316. doi: 10.3389/fimmu.2013.00316 24109481 PMC3791471

[25] Koch S Larbi A Derhovanessian E Özcelik D Naumova E Pawelec G. Multiparameter flow cytometric analysis of CD4 and CD8 T cell subsets in young and old people. Immun Ageing (2008) 5:6. doi: 10.1186/1742-4933-5-6 18657274 PMC2515281

[26] Luong Q Huang J Lee KY. Deciphering white adipose tissue heterogeneity. Biol (Basel) (2019) 8(2):23. doi: 10.3390/biology8020023 PMC6628053 30978929

[27] Bagchi DP MacDougald OA. Identification and dissection of diverse mouse adipose depots. J Vis Exp (2019) 149). doi: 10.3791/59499 PMC7017470 31355801

[28] Virtue S Vidal-Puig A. It’s not how fat you are, it’s what you do with it that counts. PLoS Biol (2008) 6(9):e237. doi: 10.1371/journal.pbio.0060237 18816166 PMC2553843

[29] Lumeng CN DelProposto JB Westcott DJ Saltiel AR. Phenotypic switching of adipose tissue macrophages with obesity is generated by spatiotemporal differences in macrophage subtypes. Diabetes (2008) 57(12):3239–46. doi: 10.2337/db08-0872 PMC2584129 18829989

[30] Hudson D Fernandes RA Basham M Ogg G Koohy H. Can we predict T cell specificity with digital biology and machine learning? Nat Rev Immunol (2023), 8:511–21. doi: 10.1038/s41577-023-00835-3 PMC9908307 36755161

[31] Jurewicz MM Stern LJ. Class II MHC antigen processing in immune tolerance and inflammation. Immunogenetics (2019) 71(3):171–87. doi: 10.1007/s00251-018-1095-x PMC6377339 30421030

[32] Prasad S Starck SR Shastri N. Presentation of cryptic peptides by MHC I is enhanced by inflammatory stimuli. J Immunol (2016) 197(8):2981–91. doi: 10.4049/jimmunol.1502045 PMC5101130 27647836

[33] Sinclair C Bains I Yates AJ Seddon B. Asymmetric thymocyte death underlies the CD4:CD8 T-cell ratio in the adaptive immune system. Proc Natl Acad Sci U S A (2013) 110(31):E2905–14. doi: 10.1073/pnas.1304859110 PMC3732981 23858460

[34] Sathaliyawala T Kubota M Yudanin N Turner D Camp P Thome JJC. Distribution and compartmentalization of human circulating and tissue-resident memory T cell subsets. Immunity (2013) 38(1):187–97. doi: 10.1016/j.immuni.2012.09.020 PMC3557604 23260195

[35] Sckisel GD Mirsoian A Minnar CM Crittenden M Curti B Chen JQ. Differential phenotypes of memory CD4 and CD8 T cells in the spleen and peripheral tissues following immunostimulatory therapy. J Immunother Cancer (2017) 5:33. doi: 10.1186/s40425-017-0235-4 28428882 PMC5394626

[36] Cibrián D Sánchez-Madrid F. CD69: from activation marker to metabolic gatekeeper. Eur J Immunol (2017) 47(6):946–53. doi: 10.1002/eji.201646837 PMC6485631 28475283

[37] Kumar BV Ma W Miron M Granot T Guyer RS Carpenter DJ. Human tissue-resident memory T cells are defined by core transcriptional and functional signatures in lymphoid and mucosal sites. Cell Rep (2017) 20(12):2921–34. doi: 10.1016/j.celrep.2017.08.078 PMC5646692 28930685

[38] Walsh DA da Silva HB Beura LK Peng C Hamilton SE Masopust D. The functional requirement for CD69 in establishment of resident memory CD8+ T cells varies with tissue location. J Immunol (2019) 203(4):946–55. doi: 10.4049/jimmunol.1900052 PMC6684481 31243092

[39] Bromley SK Akbaba H Mani V Mora-Buch R Chasse AY Sama A. CD49a regulates cutaneous resident memory CD8+ T cell persistence and response. Cell Rep (2020) 32(9):108085. doi: 10.1016/j.celrep.2020.108085 32877667 PMC7520726

[40] Richter MV Topham DJ. The α1β1 integrin and TNF receptor II protect airway CD8+ Effector T cells from apoptosis during influenza infection1. J Immunol (2007) 179(8):5054–63. doi: 10.4049/jimmunol.179.8.5054 17911590

[41] Reilly EC Lambert Emo K Buckley PM Reilly NS Smith I Chaves FA. TRM integrins CD103 and CD49a differentially support adherence and motility after resolution of influenza virus infection. Proc Natl Acad Sci U S A (2020) 117(22):12306–14. doi: 10.1073/pnas.1915681117 PMC7275699 32439709

[42] Chapman TJ Topham DJ. Identification of a Unique Population of Tissue-Memory CD4+ T cells in the Airways after Influenza Infection that is Dependent on the Integrin VLA-1. J Immunol (2010) 184(7):3841–9. doi: 10.4049/jimmunol.0902281 PMC2843798 20200271

[43] Cheuk S Schlums H Gallais Sérézal I Martini E Chiang SC Marquardt N. CD49a expression defines tissue-resident CD8+ T cells poised for cytotoxic function in human skin. Immunity (2017) 46(2):287–300. doi: 10.1016/j.immuni.2017.01.009 28214226 PMC5337619

[44] Reilly EC Sportiello M Emo KL Amitrano AM Jha R Kumar ABR. CD49a identifies polyfunctional memory CD8 T cell subsets that persist in the lungs after influenza infection. Front Immunol (2021) 12:728669. doi: 10.3389/fimmu.2021.728669 34566986 PMC8462271

[45] Mackay LK Stock AT Ma JZ Jones CM Kent SJ Mueller SN. Long-lived epithelial immunity by tissue-resident memory T (TRM) cells in the absence of persisting local antigen presentation. Proc Natl Acad Sci U S A (2012) 109(18):7037–42. doi: 10.1073/pnas.1202288109 PMC3344960 22509047

[46] Speiser DE Utzschneider DT Oberle SG Münz C Romero P Zehn D. T cell differentiation in chronic infection and cancer: functional adaptation or exhaustion? Nat Rev Immunol (2014) 14(11):768–74. doi: 10.1038/nri3740 25257362

[47] Nikolich-Žugich J. Ageing and life-long maintenance of T-cell subsets in the face of latent persistent infections. Nat Rev Immunol (2008) 8(7):512–22. doi: 10.1038/nri2318 PMC5573867 18469829

[48] Muschaweckh A Buchholz VR Fellenzer A Hessel C König PA Tao S. Antigen-dependent competition shapes the local repertoire of tissue-resident memory CD8+ T cells. J Exp Med (2016) 213(13):3075–86. doi: 10.1084/jem.2016088 PMC5154944 27899444

[49] Klenerman P Oxenius A. T cell responses to cytomegalovirus. Nat Rev Immunol (2016) 16(6):367–77. doi: 10.1038/nri.2016.38 27108521

[50] Turner SJ Doherty PC McCluskey J Rossjohn J. Structural determinants of T-cell receptor bias in immunity. Nat Rev Immunol (2006) 6(12):883–94. doi: 10.1038/nri1977 17110956

[51] Hansen SG Powers CJ Richards R Ventura AB Ford JC Siess D. Evasion of CD8+ T cells is critical for super-infection by cytomegalovirus. Science (2010) 328(5974):102–6. doi: 10.1126/science.1185350 PMC2883175 20360110

[52] Berry R Watson GM Jonjic S Degli-Esposti MA Rossjohn J. Modulation of innate and adaptive immunity by cytomegaloviruses. Nat Rev Immunol (2020) 20(2):113–27. doi: 10.1038/s41577-019-0225-5 31666730

[53] Potapov V Ong JL Langhorst BW Bilotti K Cahoon D Canton B. A single-molecule sequencing assay for the comprehensive profiling of T4 DNA ligase fidelity and bias during DNA end-joining. Nucleic Acids Res (2018) 46(13):e79. doi: 10.1093/nar/gky303 29741723 PMC6061786

[54] Ewels P Magnusson M Lundin S Käller M. MultiQC: summarize analysis results for multiple tools and samples in a single report. Bioinformatics (2016) 32(19):3047–8. doi: 10.1093/bioinformatics/btw354 PMC5039924 27312411

[55] Danecek P Bonfield JK Liddle J Marshall J Ohan V Pollard MO. Twelve years of SAMtools and BCFtools. GigaScience (2021) 10(2):giab008. doi: 10.1093/gigascience/giab008 33590861 PMC7931819

[56] Pagès H Aboyoun P Gentleman R DebRoy S. Biostrings: Efficient manipulation of biological strings. (2023), Biostrings, R package version 2.70.1, Available at: https://bioconductor.org/packages/Biostrings

[57] Gerritsen B Pandit A Andeweg AC de Boer RJ. RTCR: a pipeline for complete and accurate recovery of T cell repertoires from high throughput sequencing data. Bioinformatics (2016) 32(20):3098–106. doi: 10.1093/bioinformatics/btw339 PMC5048062 27324198

[58] Pogorelyy MV Shugay M. A framework for annotation of antigen specificities in high-throughput T-cell repertoire sequencing studies. Front Immunol (2019) 10:2159. doi: 10.3389/fimmu.2019.02159 31616409 PMC6775185

[59] Goncharov M Bagaev D Shcherbinin D Zvyagin I Bolotin D Thomas PG. VDJdb in the pandemic era: a compendium of T cell receptors specific for SARS-CoV-2. Nat Methods (2022) 19(9):1017–9. doi: 10.1038/s41592-022-01578-0 35970936

[60] Zhang H Zhan X Li B. GIANA allows computationally-efficient TCR clustering and multi-disease repertoire classification by isometric transformation. Nat Commun (2021) 12(1):4699. doi: 10.1038/s41467-021-25006-7 34349111 PMC8339063

