# Supplementary material for: Human adipose tissue as a major reservoir of cytomegalovirus-reactive T cells
Source: Front Immunol. 2023 Nov 20;14:1303724. doi: 10.3389/fimmu.2023.1303724 (PMC10694288; doi:10.3389/fimmu.2023.1303724)
Supplement: Supplementary file 2 [file Image_1.pdf]

2 Supplementary Figures and Tables

2.1 Supplementary Figures

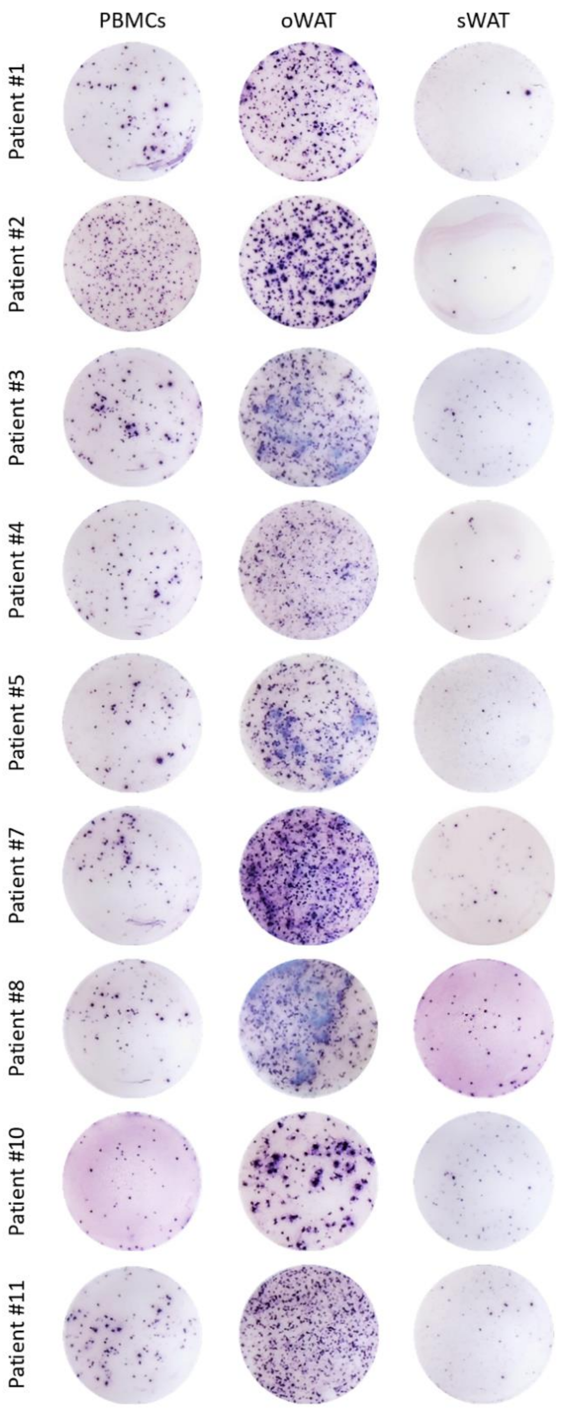

Supplementary Figure 1. Cytomegalovirus ELISpot assays in PBMCs, oWAT and sWAT of the rest of patients not shown in Figure 3.
