# Supplementary material for: Human adipose tissue as a major reservoir of cytomegalovirus-reactive T cells
Source: Front Immunol. 2023 Nov 20;14:1303724. doi: 10.3389/fimmu.2023.1303724 (PMC10694288; doi:10.3389/fimmu.2023.1303724)
Supplement: Supplementary file 3 [file Image_2.pdf]

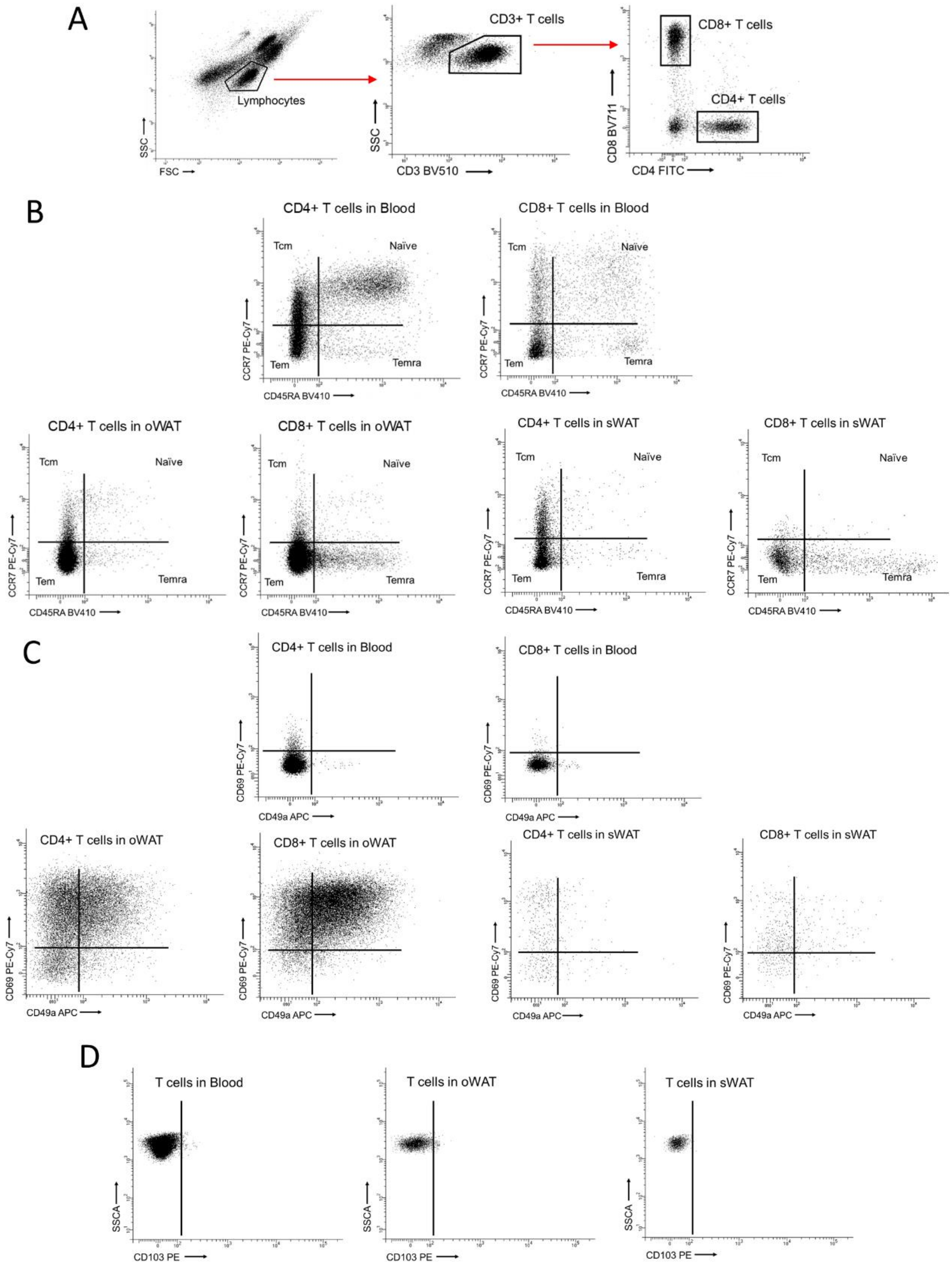

Supplementary Figure 3. Flow cytometry of blood, oWAT and sWAT samples. **(A)** SSC versus FSC plot of oWAT SVF and gating strategy to identify CD4+ and CD8+ subpopulations in Panel 2. **(B)** T cell subpopulations identified by Panel 1. **(C)** T cell subpopulations identified by Panel 2. **(D)** T cells CD103 staining.
