# Supplementary material for: Human adipose tissue as a major reservoir of cytomegalovirus-reactive T cells
Source: Front Immunol. 2023 Nov 20;14:1303724. doi: 10.3389/fimmu.2023.1303724 (PMC10694288; doi:10.3389/fimmu.2023.1303724)
Supplement: Supplementary file 4 [file Image_3.pdf]

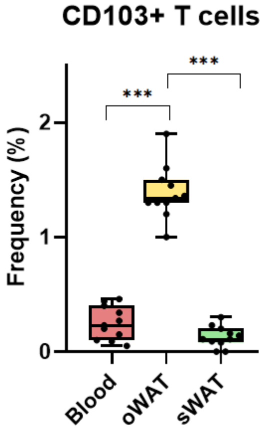

Supplementary Figure 3. Differences between the number of CD103-positive T cells in blood, oWAT and sWAT.
