# Supplementary material for: Human adipose tissue as a major reservoir of cytomegalovirus-reactive T cells
Source: Front Immunol. 2023 Nov 20;14:1303724. doi: 10.3389/fimmu.2023.1303724 (PMC10694288; doi:10.3389/fimmu.2023.1303724)
Supplement: Supplementary file 5 [file Presentation_1.pdf]

Supplementary Material

1 Supplementary Protocol 1. TCRβ library preparation methodology.

Here, we detailed the process of the TCRβ library preparation for Next Generation Sequencing (Figure 1A of Supplementary Protocol 1). All the steps are carried out with *Pfu* DNA polymerase due to its low error rate (1-2 × 10^{-6}) (1) and high thermal stability. All oligonucleotides and templates (Table 1 of Supplementary Protocol 1) used in the procedure were manufactured by IDT (Integrated DNA Technologies). Details on PCRs conditions are shown in Table 2 of Supplementary Protocol 1. The process is detailed in 5 stages as follows:

1. Functionalization of magnetic particles.

We used Sera-MagTM Magnetic Carboxylate-Modified Microparticles (MG-CM) from Cytiva, which are uniform, colloidally stable, monodispersed, non-porous spheres, and have 1μm diameter. Their nucleus comprises 40% of magnetite (Fe3O4) and is encapsulated by proprietary polymers. Stability is maintained by negatively charged carboxylic groups on their surface ready to covalently couple amino groups. They are appropriate to work with because of their higher magnetism, mobility, low sedimentation rate, and compatibility with high temperatures and extreme pH.

In order to functionalize the microparticles. oligonucleotides (0.5pmoles per µg of particles) were coupled via a 5' amino (NH2) modification with EDAC (1-ethyl-3-(3-dimethylaminopropyl) carbodiimide hydrochloride) to establish an amide bond with the carboxylic groups. The reaction was performed during 4-5h with continuous rotation in 1M NaCl. 100 mM MES (2-(N-morpholino) ethanesulfonic acid) at pH 5. Oligonucleotides included the sequence of adaptor A, one barcode, and a complementary sequence to segment C at its 3' end.

2. Enrichment PCR

The first step was a multiplex PCR. We designed 48 forward primers which simultaneously amplify every V segment (54 functional segments and 11 pseudogenes), and one reverse primer common for the 2 C segments (Table 1). The collection of forward primers was designed over a highly conserved region in the 5'-UTR of V segments (near the START codon). These oligonucleotides show an elevated homology in their 3' end sequences. which enhance the control over the polymerization of primers during the enrichment PCR.

3. "Fishing" PCR

12µl of functionalized particles were added to the product of the multiplex PCR and 3 additional PCR cycles were performed. The particle-bounded oligonucleotides, acting as nested reverse primers, elongated and acquired the reverse sequence of the TCRβ population. leaving behind all waste PCR products.

After this “fishing” PCR, magnetic particles were sedimented on a magnet and washed in 200mM NaOH to remove any unbound DNA strands. Two additional washes in TRIS-HCl 200mM pH 7.4 equilibrated pH. Lastly, particles were resuspended in TRIS-HCl 50mM pH 7.4.

4. Extension of the P1 adaptor

By using a collection of 48 templates, the single-stranded DNA (bounded to particles) acquired the P1 adaptor sequence at its 3’ end. This process was patented (PCT/ES2019/070494) as a replacement for the ligation reaction. Templates consisted of the sequences of each multiplex primer at its 3’ end and the sequence of the P1 adaptor at its 5’ end (Table 1). Moreover, an inverted thymine at the 3’ end was added to the templates to avoid their elongation. By an “extension” PCR of 3 cycles, the single-stranded DNA completed the P1 adaptor elongating towards 5'. Finally, an alkaline washing (identical to the previously described) eliminated the collection of templates.

5. Final PCR

The final step is a simple PCR with a pair of primers, each of them against one of the adaptors, to release the DNA of interest from the microparticles. After this last PCR, there is a purification step with ProNex® Size-Selective Purification System from Promega following manufacturer instructions. The final product includes amplicons with the structure depicted in Figure 1B of Supplementary Protocol 1.

References

1. McInerney P, Adams P, Hadi MZ. Error Rate Comparison during Polymerase Chain Reaction by DNA Polymerase. Mol Biol Int. 2014;2014:287430.

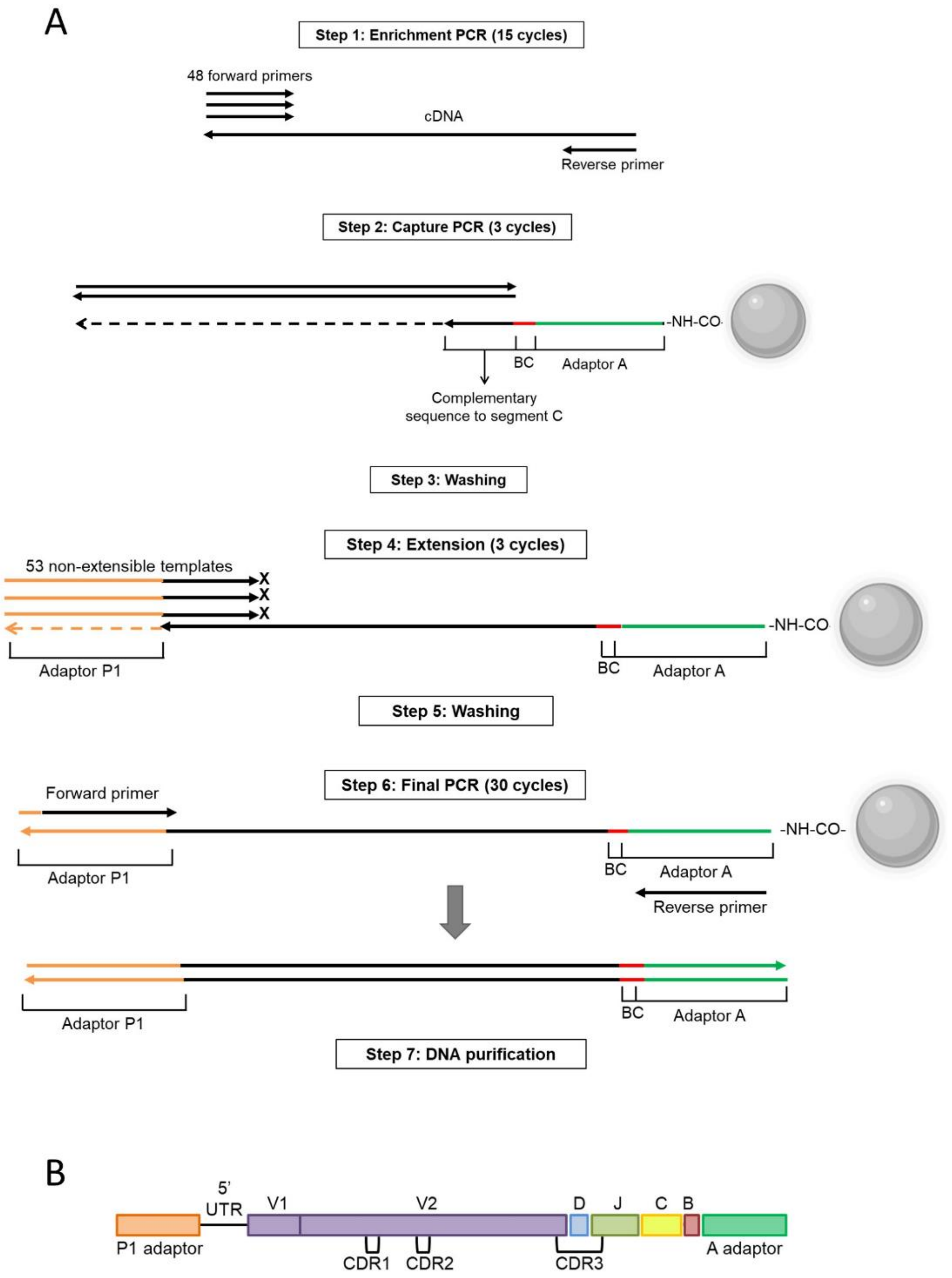

Figure 1. Overview of TCRβ library preparation. **(A)** Steps for the library preparation methodology. **(B)** Structure of the final amplicons of the library preparation. BC. barcode; UTR. untranslated region; CDR. complementary determining region; V. variable; D. diversity; J. join; C. constant.

Table 1. List of oligonucleotides/templates for the TCR library preparation.

	Forward oligonucleotide(s)/templates		Reverse oligonucleotide	
**Stage**	**Name**	**Sequence**	**Name**	**Sequence**
**Enrichment PCR**	1.1-F	atcctgtagcacctgccat	Mx-R	tgtgggagatctctgcttctgttggctcaaa
	1.2-F	gtcctggagcacctgccat		
	1.3-F	atcctgcagttcctgccat		
	1.4-F	tcctgcagcccctgccat		
	1.5-F	cctg**B**cctgaccctgccat		
	1.6-F	ccttccctgaccctgccat		
	1.7-F	ctggcctgaccctgccat		
	1.8-F	agctaggagatcctgccat		
	1.9-F	cctgctgtgatcctgccat		
	1.10-F	ggggtggtattcctgccat		
	2.1-F	cctgccccactgtgccat		
	2.2-F	tcctgtccctctgtgccat		
	2.3-F	cctggcccactgtgccat		
	2.4-F	gcctgccccattgtgccat		
	3.1-F	gtctgcctcactctgccat		
	3.2-F	tctgctctcactctgccat		
	3.3-F	acctgccccaccctgccat		
	3.4-F	cccccagctccttggctat		
	4.1-F	tgtcttgtagcatctgccat		
	4.2-F	cgtcttgcagcatctgccat		
	5-F	ctagccccaactgtgccat		
	6-F	ctccatcctgcctcttcat		
	7-F	ttccttcttcaaagcagccat		
	8-F	ggctgctctgctgtgcgg		
	9.1-F	tcctgccctggagctgaaa		
	9.2-F	tcctggcctggacctgaaa		
	10.1-F	cctgccctgactctgtcat		
	10.2-F	cctggtctgacactgtcat		
	11.1-F	gaaggtggtgtgaggccat		
	11.2-F	ttctcaggggagaggccat		
	12-F	cactgcctgacctcaccat		
	13-F	caccaggctcctctgccat		
	14-F	gggcaccaggctcctcgg		
	15-F	tttacctgggtcctgccat		
	16-F	cctgtgggctccctccat		
	17-F	aaggcccccattgcactat		
	18-F	cccagactagctgaaggaaa		
	PG1.1-F	tccactgtggtgtggtccat		
	PG3.2-F	gctgcaggctcctctgctat		
	PG5.2-F	gcctgctacgctgtgccat		

**Enrichment PCR**	PG7.5-F	cctggcctgaccctgccat		
	PG8.2-F	ctcactctgaccctaccat		
	PG12.1-F	ttctgctctcactctgccat		
	PG12.2-F	atctgttctcactctgccat		
	PG16-F	tcttcccctaattctgccat		
	PG21.1-F	cttctctgctgtgtggccat		
	PG22.1-F	gagctgggtcctctgctat		
	PG26-F	cttctctgctgtgtgatcat		
**“Fishing PCR”**	-	-	A-BCX-R	5' Amino Modifier C12-ccatctcatccctgcgtgtctccgactcagXXXXXXXXXcgatcagcgacctcgggtgggaaca
**P1 extension**	1.1-P1	ctacgcctccgctttcctctctatgggcagtcggtgatatcctgtagcacctgccat	-	-
	1.2-P1	ctacgcctccgctttcctctctatgggcagtcggtgatgtcctggagcacctgccat		
	1.3-P1	ctacgcctccgctttcctctctatgggcagtcggtgatatcctgcagttcctgccat		
	1.4-P1	ctacgcctccgctttcctctctatgggcagtcggtgattcctgcagcccctgccat		
	1.5-P1	ctacgcctccgctttcctctctatgggcagtcggtgatcctg**B**cctgaccctgccat		
	1.6-P1	ctacgcctccgctttcctctctatgggcagtcggtgatccttccctgaccctgccat		
	1.7-P1	ctacgcctccgctttcctctctatgggcagtcggtgatctggcctgaccctgccat		
	1.8-P1	ctacgcctccgctttcctctctatgggcagtcggtgatagctaggagatcctgccat		
	1.9-P1	ctacgcctccgctttcctctctatgggcagtcggtgatcctgctgtgatcctgccat		
	1.10-P1	ctacgcctccgctttcctctctatgggcagtcggtgatggggtggtattcctgccat		
	2.1-P1	ctacgcctccgctttcctctctatgggcagtcggtgatcctgccccactgtgccat		
	2.2-P1	ctacgcctccgctttcctctctatgggcagtcggtgattcctgtccctctgtgccat		
	2.3-P1	ctacgcctccgctttcctctctatgggcagtcggtgatcctggcccactgtgccat		
	2.4-P1	ctacgcctccgctttcctctctatgggcagtcggtgatgcctgccccattgtgccat		
	3.1-P1	ctacgcctccgctttcctctctatgggcagtcggtgatgtctgcctcactctgccat		
	3.2-P1	ctacgcctccgctttcctctctatgggcagtcggtgattctgctctcactctgccat		
	3.3-P1	ctacgcctccgctttcctctctatgggcagtcggtgatacctgccccaccctgccat		
	3.4-P1	ctacgcctccgctttcctctctatgggcagtcggtgatcccccagctccttggctat		
	4.1-P1	ctacgcctccgctttcctctctatgggcagtcggtgattgtcttgtagcatctgccat		
	4.2-P1	ctacgcctccgctttcctctctatgggcagtcggtgatcgtcttgcagcatctgccat		
	5-P1	ctacgcctccgctttcctctctatgggcagtcggtgatctagccccaactgtgccat		
	6-P1	ctacgcctccgctttcctctctatgggcagtcggtgatctccatcctgcctcttcat		
	7-P1	ctacgcctccgctttcctctctatgggcagtcggtgatttccttcttcaaagcagccat		
	8-P1	ctacgcctccgctttcctctctatgggcagtcggtgatggctgctctgctgtgcgg		
	9.1-P1	ctacgcctccgctttcctctctatgggcagtcggtgattcctgccctggagctgaaa		
	9.2-P1	ctacgcctccgctttcctctctatgggcagtcggtgattcctggcctggacctgaaa		
	10.1-P1	ctacgcctccgctttcctctctatgggcagtcggtgatcctgccctgactctgtcat		
	10.2-P1	ctacgcctccgctttcctctctatgggcagtcggtgatcctggtctgacactgtcat		
	11.1-P1	ctacgcctccgctttcctctctatgggcagtcggtgatgaaggtggtgtgaggccat		

**P1 extension**	11.2-P1	ctacgcctccgctttcctctctatgggcagtcggtgatttctcaggggagaggccat	-	-
	12-P1	ctacgcctccgctttcctctctatgggcagtcggtgatcactgcctgacctcaccat		
	13-P1	ctacgcctccgctttcctctctatgggcagtcggtgatcaccaggctcctctgccat		
	14-P1	ctacgcctccgctttcctctctatgggcagtcggtgatgggcaccaggctcctcgg		
	15-P1	ctacgcctccgctttcctctctatgggcagtcggtgattttacctgggtcctgccat		
	16-P1	ctacgcctccgctttcctctctatgggcagtcggtgatcctgtgggctccctccat		
	17-P1	ctacgcctccgctttcctctctatgggcagtcggtgataaggcccccattgcactat		
	18-P1	ctacgcctccgctttcctctctatgggcagtcggtgatcccagactagctgaaggaaa		
	PG1.1-P1	ctacgcctccgctttcctctctatgggcagtcggtgattccactgtggtgtggtccat		
	PG3.2-P1	ctacgcctccgctttcctctctatgggcagtcggtgatgctgcaggctcctctgctat		
	PG5.2-P1	ctacgcctccgctttcctctctatgggcagtcggtgatgcctgctacgctgtgccat		
	PG7.5-P1	ctacgcctccgctttcctctctatgggcagtcggtgatcctggcctgaccctgccat		
	PG8.2-P1	ctacgcctccgctttcctctctatgggcagtcggtgatctcactctgaccctaccat		
	PG12.1-P1	ctacgcctccgctttcctctctatgggcagtcggtgatttctgctctcactctgccat		
	PG12.2-P1	ctacgcctccgctttcctctctatgggcagtcggtgatatctgttctcactctgccat		
	PG16-P1	ctacgcctccgctttcctctctatgggcagtcggtgattcttcccctaattctgccat		
	PG21.1-P1	ctacgcctccgctttcctctctatgggcagtcggtgatcttctctgctgtgtggccat		
	PG22.1-P1	ctacgcctccgctttcctctctatgggcagtcggtgatgagctgggtcctctgctat		
	PG26-P1	ctacgcctccgctttcctctctatgggcagtcggtgatcttctctgctgtgtgatcat		
**Final PCR**	Final-F	ccactacgcctccgctttcctctctatgg	Final-R	ccatctcatccctgcgtgtctccgactca

B=G +T + C. Xs in red simulate the barcode sequence. In green. is the sequence of the A adaptor. In orange. sequences elongated of the P1 adaptor

Table 2. Conditions for the different PCRs of the library preparation for TCRβ repertoire.

	Enrichment PCR	"Fishing" PCR	P1 extension	Final PCR
**Forward primer/s**	75nM	None	75nM	200nM
**Reverse primer**	150nM	Microparticles	None	200nM
**Microparticles**	-	12µl	Microparticles after a washing step	Microparticles after a washing step
**Total volume**	20 µl	100µl	100 µl	30 µl
**Cycles**	15	3	3	40
**Annealing temperature (time)**	62˚C (30'')	68˚C (1')	63˚C (3')	65˚C (30")
**Elongation temperature (time)**	74˚C (2')	74˚C (2')	74˚C (5')	74˚C (2')
**Amplicon length**	~550 bp	~500 bp	~550 bp	~550 pb

The amplicon length changes depending on the V segment and CDR3 composition
