# Supplementary material for: Human adipose tissue as a major reservoir of cytomegalovirus-reactive T cells
Source: Front Immunol. 2023 Nov 20;14:1303724. doi: 10.3389/fimmu.2023.1303724 (PMC10694288; doi:10.3389/fimmu.2023.1303724)
Supplement: Supplementary file 6 [file Table_1.pdf]

2.2 Supplementary tables

Supplementary Table 1. HLA Genotyping of patients.

	HLA-I genotyping					
**Patient**	**HLA-A**		**HLA-B**		**HLA-C**	
**1**	A*66:01	A*68:01	B*15:01	B*44:03	C*03:04	C*16:01
**2**	A*01:01	A*31:01	B*07:02	B*67:01P	C*07:02	C*12:03
**3**	A*02:02	A*03:01	B*41:01	B*57:01	C*06:02	C*17:01
**4**	A*29:02	A*30:04	B*44:03	B*53:01	C*15:05	C*16:01
**5**	A*02:01	A*02:01	B*18:01	B*56:01	C*01:02	C*04:01
**6**	A*02:01	A*29:02	B*35:01	B*57:01	C*04:01	C*07:01
**7**	A*01:01	A*25:01	B*08:01	B*18:01	C*07:01	C*12:03
**8**	A*02:01	A*02:01	B*18:01	B*38:01	C*07:01	C*12:03
**9**	A*11:01	A*29:02	B*44:03	B*52:01	C*12:02	C*16:01
**10**	A*30:02	A*33:01	B*14:02	B*18:01	C*05:01	C*08:02
**11**	A*01:01	A*29:02	B*37:01	B*44:03	C*06:02	C*16:01
	**HLA-II genotyping**					
**Patient**	**HLA-DRB1**		**HLA-DPB1**		**HLA-DQB1**	
**1**	DRB1*04:01	DRB1*07:01	DPB1*03:01P	DPB1*04:01	DQB1*02:02	DQB1*03:02
**2**	DRB1*14:01P	DRB1*15:01	DPB1*02:01	DPB1*04:01	DQB1*05:03	DQB1*06:02
**3**	DRB1*07:01	DRB1*11:02	-	-	DQB1*03:03	DQB1*03:19
**4**	DRB1*13:02	DRB1*16:01	DPB1*02:01P	DPB1*17:01P	DQB1*05:02	DQB1*06:04
**5**	DRB1*01:01	DRB1*11:04	DPB1*04:01	DPB1*06:01	DQB1*03:01	DQB1*05:01
**6**	DRB1*04:01	DRB1*07:01	DPB1*04:01	DPB1*04:02	DQB1*02:02	DQB1*03:02
**7**	DRB1*03:01	DRB1*15:01	DPB1*03:01P	DPB1*23:01	DQB1*02:01	DQB1*06:02
**8**	DRB1*04:05	DRB1*13:01	DPB1*03:01P	DPB1*105:01	DQB1*03:02	DQB1*06:03
**9**	DRB1*07:01	DRB1*15:02	DPB1*02:01	DPB1*11:01	DQB1*02:02	DQB1*06:01
**10**	DRB1*01:02	DRB1*03:01	DPB1*04:01	DPB1*15:01	DQB1*02:01	DQB1*05:01
**11**	DRB1*01:03	DRB1*03:01	DPB1*01:01	DPB1*04:02	DQB1*02:01	DQB1*03:01
